# Towards a software architecture to manage occupational safety at grain handling and storage facilities

**DOI:** 10.1038/s41598-022-06534-8

**Published:** 2022-02-16

**Authors:** Sabrina Dalla Corte Bellochio, Paulo Carteri Coradi, Vinícius Maran, Marcos Alves dos Santos, Luan Willig Silveira, Paulo Eduardo Teodoro

**Affiliations:** 1 Department of Agricultural Engineering, Postgraduate at Agricultural Engineering, Federal University of Santa Maria, Avenue Roraima, 1000, Camobi, Santa Maria, RS 97105900 Brazil; 2 Laboratory of Postharvest-Research Group at Postharvest Innovation: Technology, Quality and Sustainability, Campus Cachoeira do Sul, Federal University of Santa Maria, Taufik Germano, 3013, Passo D’Areia, Cachoeira Do Sul, RS 96506-322 Brazil; 3 Laboratory of Ubiquitous, Mobile and Applied Computing (LUMAC), Campus Cachoeira do Sul, Federal University of Santa Maria, Taufik Germano, Passo D’Areia, Cachoeira Do Sul, RS 301396506-322 Brazil; 4 Department of Agronomy, Campus de Chapadão Do Sul, Federal University of Mato Grosso Do Sul, Chapadão Do Sul, MS 79560-000 Brazil

**Keywords:** Environmental sciences, Health occupations, Risk factors, Engineering, Mathematics and computing

## Abstract

The study had as objective to evaluate occupational hazards on grain storage unit to define a conceptual model, implemented in an algorithm to manage the grains storage facilities safety standards compliance. Sampling points location were defined for static quantification of noise, dust and heat stress hazards in grains pre-processing operations to indicate the effectiveness of the control measures implemented. Safety standards applied to grain handling and storage facilities were identified and selected. Chart flows were elaborated to the algorithm logics and conceptual modeling. The highest level of noise was present in the grain cleaning operation (99.1 dB), while the expedition operation has the highest level of dust (20.27%). The heat stress was present in the grain drying operation (43.64 WBGT). Noise analysis did not show a difference between grains, only between operations. The flow of corn grain mass caused higher dust concentrations in the expedition operation. The method applied to characterize and quantify the hazards in grain storage units was satisfactory, and it is recommended as standard, for use in corn and soybean grains handling and storage units. The algorithm to manage occupational safety at storage facilities collaborates to monitor the safety compliance on postharvest operations.

## Introduction

Agriculture activity is considered one of the most hazardous occupations^1^. Many incidents and occupational diseases on agriculture are reported by workers of postharvest operations at grain storage facilities. The occupational hazards at grain storage units are generated due the post-harvest operations comprised, in general, by receiving the grains, pre-cleaning, drying, cleaning, storage and shipping^2,3^.

Confined space^4–7^ work at height^8^, grain entrapment^9–12^, machine entanglement^13,14^, fire and explosions^15,16^, ergonomics^17,18^, besides noise^19^, dusts^20^ and heat-induced illness^21^ are common hazards at grain storage facilities^22,23^.

Activities of grains handling, cleaning and shipping generates dusts in the grain storage unit^24–27^. Machinery and equipment operation, such ones used at grains reception and unloading, dryer’s operation, grains transport and handling, generates occupational noise^28^ and, especially the furnace on grain drying process, offers heat hazard at the activity of feeding and maintaining the fire^29^.

To know and manage the occupational hazards in the work environment is fundamental in preserving the worker's health. Thus, one of the requirements of the occupational health and safety management system is compliance with the national standards established for grain handling^30^. In Brazil, the legislation related to the theme refers mainly to the Regulatory Norms, instituted by Ordinance 3,214 of the Ministry of Labor, in 1978. The Regulatory Norms consist of obligations, rights and duties that must be fulfilled by employers and workers, with the objective to improve the occupational safe and healthy, preventing the occurrence of illnesses and incidents at work^31^.

Compliance with legal requirements is the basis for preventing incidents^32^ and occupational illnesses^33^, because compliance with the legislation results in the improvement of occupational health and safety^34^. However, to manage the legal compliance, especially in small and medium-sized businesses, is not a simple task, as it is essential that organizations have access to tools and methodologies that provide the necessary knowledge about health and safety standards^35^.

The agribusiness value highlights the emphasis on actions aimed to prevent incidents and occupational diseases. In that way, to identify, evaluate and quantify occupational hazards at grain storage facility is fundamental. Given the importance of postharvest processes in agriculture, the objective of this study was to quantify and evaluate noise, dusts and heat occupational hazards on grain storage unit at unloading, cleaning, drying and expedition processes and compare results between operations. Besides that, to define a conceptual model, that was used to define software architecture to manage the grains storage facilities safety standards compliance.

## Subjects and methods

### Grain storage unit characterization and definition of the sampling points

The data were extracted from a Collector type Storage Unit. In general, these kinds of storage unit receives the grains directly from the crops and provide pre-processing and storage services to various producers. In addition to receiving grains from different producers, this type of storage unit has the characteristic of containing throughout the harvest, a large flow of grains on the different pre-processing operations. The high grain flow can increase the occupational risks with dust, noise and heat.

**Figure 1:**
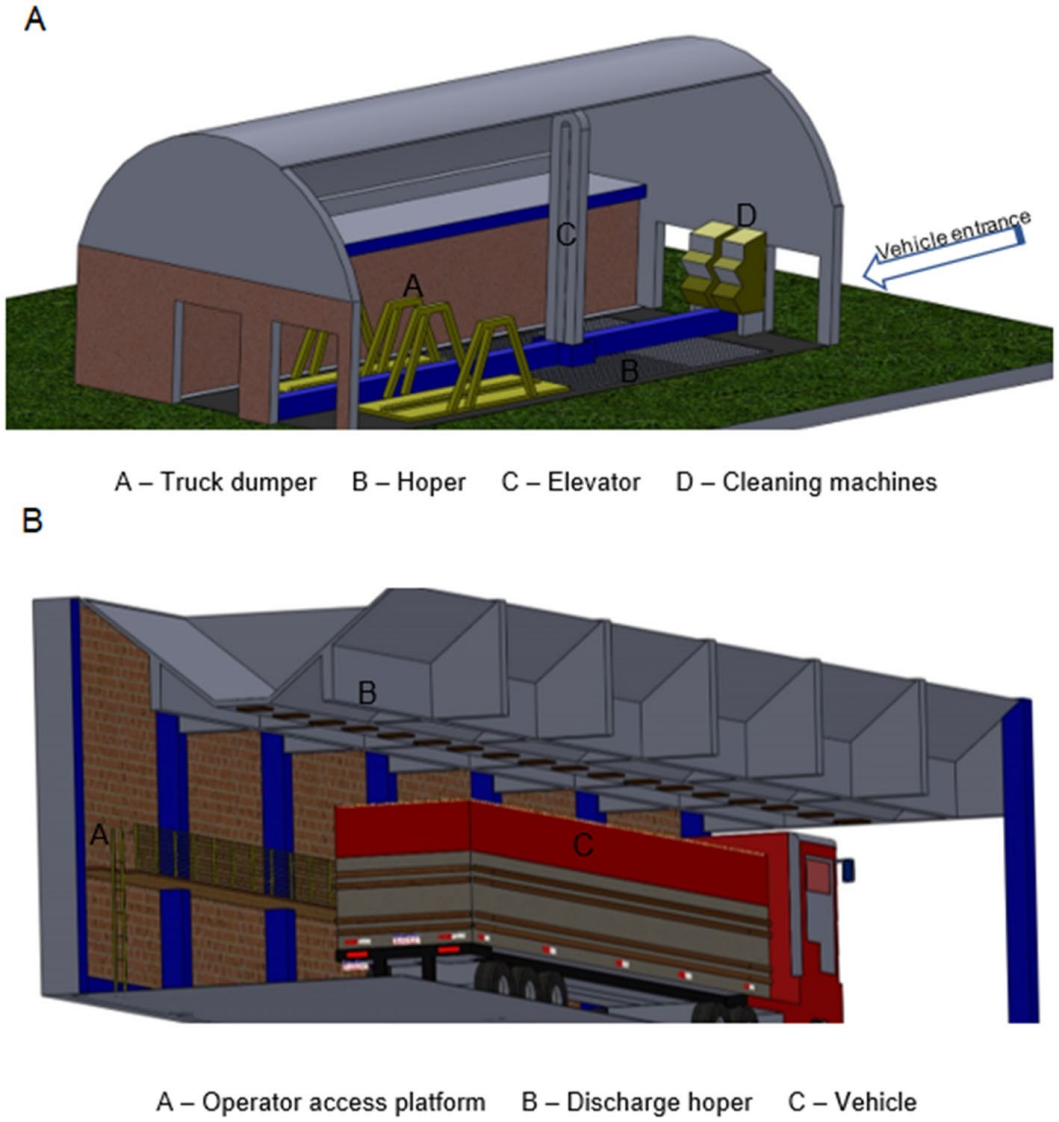
Representation of the grains unloading and cleaning area (**A**), representation grains expedition area (**B**).

The grain storage unit pre-processes and stores grains of soybean and corn in bulk. The structure has operations for grains receiving, pre-cleaning, drying, cleaning, storage and shipping (Fig. 1A–B).

### Static reference sampling points

The sampling points determined for the application of the methodologies for quantifying occupational hazards in the environment, aimed to represent centralization in the environments for receiving, cleaning and expediting grains, which have circulation of workers in the execution of their tasks.

In addition, the proposed configuration of points makes it possible to capture the noise distribution in the discharge and in the loading of grains in the transport vehicle, in a uniform way, allowing to identify the stages of the operation that present greater noise intensity.

### Grains unload

The noise quantification in the grain unloading operation was carried out at the rear of the vehicle in unloading by means of hydraulic tipping equipment, 2 m from its rear right side, at a height of 1.6 m in relation to the ground.

For the dust quantification in the grain unloading operation, the equipment was positioned at a height of 1.6 m in relation to the ground, centralized in relation to the vehicle being unloaded and 3 m away from the rear of the vehicle. This positioning allows sampling of the dust concentration in the surroundings of the operation. The access gates to the environment remained open.

### Grains cleaning

The noise sampling points in the cleaning operation included the positions corresponding to the monitoring, inspection, adjustments and collection of waste. Thus, the quantification occurred on the elevated platform of cleaning machine operation, at 1.6 m height in relation to the platform, in front of it and on its sides, at a height of 1.6 m in relation to the ground.

In the cleaning operation, the dust sampling equipment was positioned at a height of 1.6 m in relation to the ground and 2 m away from the front of the cleaning machines, in a centralized region, in relation to the machines.

### Grains drying

The heat stress measuring equipment was installed in front of the furnace that is manually supplied with firewood, distant from this 1.2 m. The installation height of the equipment was 1.2 m, which corresponds to the height of the furnace feeding opening and the height of the operator's chest. There were no obstacles between the equipment and the furnace.

### Grains expedition

The noise quantification in the grain expedition operation took place on the platform where the operator performs the opening of the discharge bins by hatches, at a height of 1.6 m in relation to the platform. The sampling took place in 3 different positions corresponding to the initial, middle and final portions of the vehicle body being loaded. In the grain expedition operation, the dust sampling pump was positioned on the platform where the operator opens the discharge bins by hatches, at a height of 1.6 m in relation to the platform. The position was central in relation to the open spouts.

### Occupational hazards

The methodology described for the occupational noise, dust hazards and heat stress quantification are described by Occupational Hygiene Standards NHO 01^36^, NHO 08^37^ and NHO 06^38^, respectively. The differential of this study is to approach the application of these methodologies, for the static environmental quantification of occupational hazards of noise, dust and heat stress in the grain storage unit and the definition of the critical points of sampling by grains unloading, cleaning and shipping. In this way, the proposed standard may be employed in the evaluation of grain storage units, in pre-processing and storage operations, in order to determine the effectiveness of hazards control measures.

### Noise

The occupational noise quantitative assessment was performed using a digital sound meter IP-170L (Impac Comercial e Tecnologia Ltda, Butantã, São Paulo, Brazil) with a sponge protector on the microphone; and calibrator SC-942 (Lutron Electronic Enterprise Co., Ltd., Taipei, Taiwan). The noise measurement equipment was adjusted for operation in the “A” compensation circuit and slow response (slow), for the quantification of the equivalent continuous noise level (LEQ) in 10 seconds^39^. The methodology applied for determining occupational noise follows the technical procedure determined by NHO 01^36^. The evaluations were static, with reference to the height of 1.6 m above the floor surface, with the equipment positioned on a tripod. The readings were performed during the workday, with the equipment facing the noise generating source.

**Figure 2:**
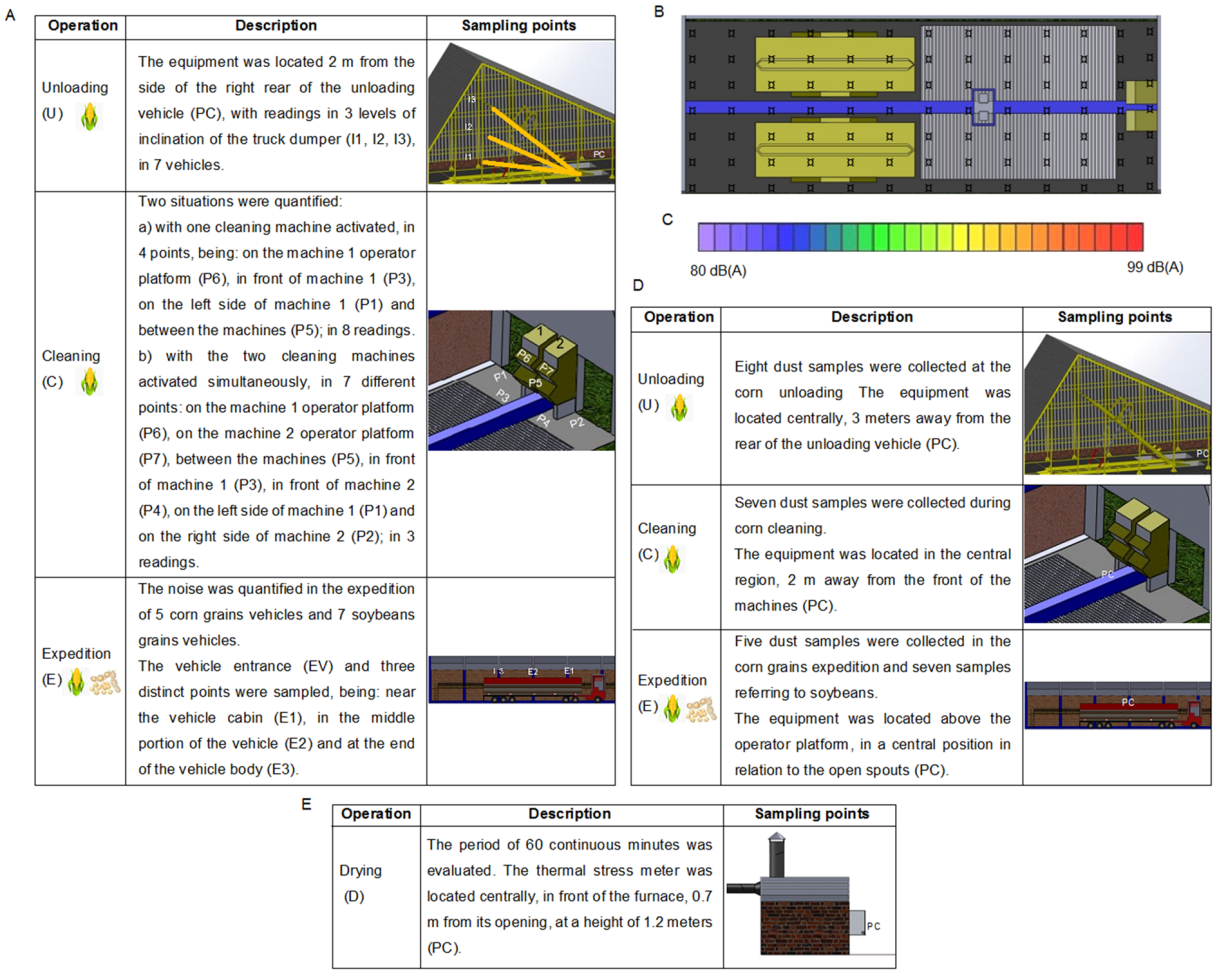
Representation of noise data collection points (**A**), noise collection points in top view of the grain receiving and cleaning area (**B**), noise colorimetric scale (**C**), representation of dust data collection points (**D**), representation of heat stress data collection points (**E**).

Occupational noise was quantified in the corn grains mechanized unloading operation, by pneumatic truck dumpers, corn grains pre-cleaning and/or cleaning operation and in the corn and soybean expedition operation, with 3 repetitions of each reading. In the grain unloading operation, the equipment was positioned 2 m away, on the right side of the rear of the unloading vehicle. From the moment the vehicle is lifted through the use of the tipper, three moments of collection are performed: at the beginning of the elevation (I1), when the first grains begin to fall; in the mean tilt position (I2); and, finally, in the maximum elevation position (I3), when the last grains leave the vehicle (Fig. 2A).

For the grains cleaning operation, the sampling points included the positions around and in front of the machine, in addition to the operator platform. These points are used for monitoring, inspection and adjustments of the machine and also for waste collection. It is important to quantify when only one machine is in operation, due to the smaller flow of grains in pre-processing and when all machines are in joint operation, as the noise is not added, as it is expressed in a logarithmic function.

In the grain expedition operation, three points were determined, from the platform that the operator performs the opening of the discharge spouts, these being: in the initial portion of the vehicle body (E1), closest to the vehicle cabin; in the vehicle's average body position (E2); and, at the end of the vehicle body (E3). This configuration of noise quantification points makes it possible to capture the grain load distribution uniformly in the transport vehicle.

The tolerance limit and the action level defined by NR 15^40^ were considered. The statistical analysis used was the Student T test^29^ to check the differences between operations (unloading, cleaning with one machine and cleaning with two machines and shipping) and types of grain (corn and soybeans). Lower Confidence Limit (LIC) and Upper Confidence Limit (LSC). Noise quantification was performed for operations related to bulk corn and soybean, in the same storage unit, being in the months of August and September 2019 for soybean and in January 2020 for corn.

### Noise map

The noise map for the built area that houses the receiving operation and grains pre-cleaning and/or cleaning was elaborated. For this, its space was divided into equidistant points, every 2.5 m, forming a square mesh, where the sound pressure level readings were carried out, with three repetitions per point^41^. The points were marked on the floor, with chalk, for the best visualization during data collection (Fig. 2B).

By means of computational modeling, using the Surfer® software, with a superimposed image of the 2D plan, elaborated in AutoCAD®, the equipment readings were launched, according to the Cartesian coordinates of each point in which the noise was quantified. The colorimetric scale corresponding to the values of 80 dB (A) to 99 dB (A) was used, with blue tones for lower noise intensities and red tones for higher intensities (Fig. 2C).

The noise readings were executed in two conditions, generating a noise map for each condition. The first evaluation considers only one cleaning machine activated and the second evaluation comprises two machines in operation at the same time.

### Dusts

The grain dust quantification was performed in the operations of corn grains unloading in the hopper, corn grains cleaning and corn and soybean grains expedition. Samples for the quantitative assessment of total dust were carried out on random days, over the period of corn receiving and cleaning and the soybean and corn expedition.

A sampling pump Gilair 34 (Sensidyne Industrial Health & Safety Instrumentation, St. Petersburg, Florida, USA) and flow calibrator 4146 (TSI Incorporated, Shoreview, Minnesota, USA) were used collection device and hoses. The sampling pump was calibrated for a flow rate of 1.5 L/min and positioned by means of a metallic support, at a height of 1.6 m from the floor. Static collection was performed, with the equipment being positioned at a fixed point, in order to determine the effectiveness of the control measures.

To determine the total dust, the technical procedure determined by NHO 08 was followed^37^. The time taken to collect the dust samples in the grain unloading process at the hopper corresponded to the grains unloading from one vehicle; in the grains cleaning process, the collection was 30 min, related to approximately two charges; and, in the grain expedition process, it corresponded to the loading of grains in one vehicle.

As for the collection points, for the grains unloading, the sampling pump was positioned centrally, 3 m away from the rear of the unloading vehicle. In the grains cleaning operation, the equipment was positioned 2 m away from the front of the cleaning machines, in the central region.

Finally, in the grain expedition process, the sampling pump was positioned on the platform where the operator performs the opening of the discharge spouts, in a central position in relation to the open spouts (Fig. 2D). This configuration of dust quantification points makes it possible to capture the dispersion of dust around the operation being performed.

The samples analysis laboratory was Solutech Chemical Analysis, which makes use of the gravimetric method, given by NHO 03 and NIOSH 0500^42^. The laboratory sent the filter holders (cassettes), with a flow rate from 1 L / min to 2 L / min, identified, with the respective PVC membrane filters of 5 μm pore, 37 mm in diameter and 4 mm diameter orifice for the entry of air, pre-weighed.

The results obtained, by process and by product, were treated statistically^43^, recommended by the National Institute for Occupational Safety and Health (NIOSH) and the American Industrial Hygiene Association (AIHA), and compared to the tolerance limit established by the American Conference of Governmental Industrial Hygienists (ACGIH), for total cereal dust and particles not otherwise specified (PNOS).

### Heat

The quantitative assessment of the occupational heat stress generated by the furnace was carried out using the stress thermal meter Questemp 34 (TSI Incorporated, Shoreview, Minnesota, USA) consisting of a globe thermometer, a natural wet bulb thermometer and a dry bulb thermometer. The methodology applied for determining heat follows the technical procedure determined by NHO 06^38^. The thermal stress meter was previously calibrated and installed on a telescopic tripod, 0.7 m away from the front of the furnace. The equipment height corresponding to the furnace opening and operator chest of 1.2 m, without the presence of obstacles between the equipment and the heat source (Fig. 2E). The natural wet bulb thermometer reservoir was filled with distilled water and the cotton wick was immersed.

The temperature readings were taken after the passage of 20 min, considering the installation of the device in the environment, to stabilize the sensors. The furnace door remained closed during the firing and was only opened for refueling. Thus, it remained open for 10 min, in order to allow stabilization of the measurement set and data collection. For this, the sensor bar was removed from the instrument and positioned on the tripod, the instrument being handled by means of a remote cable at a distance of 3 m.

The period of 60 continuous minutes was evaluated, corresponding to the most unfavorable thermal overload condition during the working day, covering the entire exposure cycle. A digital stopwatch was used to determine the length of stay of the worker in each thermal situation. The duration of each identified physical activity was determined by timings, obtained by observing the worker activities performance.

As in most storage units where the drying process uses a furnace, it is manually supplied with wood and the activity includes the search for combustible material in an open environment. Thus, the activities performed by the worker correspond to the furnace supply; followed by a period of rest and rehydration; the grains sampling in the drying process, both in the same environment where the furnace is located; and the search for firewood, which consists of bringing it in a wheel hand cart from an external environment, with solar charge, to the side of the furnace.

The equipment data from the readings and the timing of time spent by the worker in different thermal situations and in the physical activities performed were recorded. The collection was carried out in January, corresponding to the summer season. The Wet Bulb Globe Thermometer Index (WBGT) was calculated for each thermal situation, without the presence of solar charge and with the presence of solar charge, using Eqs. 1 and 2, respectively:

1 $${\text{WBGT }} = \, 0.{7}\,T_{w} + \, 0.{3}\,T_{g}$$

2 $${\text{WBGT }} = \, 0.{7}\,T_{w} + \, 0.{1}T + \, 0.{2}\,T_{g}$$

where *T*_*w*_: wet-bulb temperature (in Celsius), *T*_*g*_: globe thermometer temperature (in Celsius), *T*: temperature (in Celsius).

Using the weighted average of the thermal situations identified in the exposure cycle, an average WBGT was calculated. In addition, were taken in consideration the limits of exposure to heat stress by NR 15, which observe the metabolic rate of the activity and the calculated WBGT^40^.

### Statistical multivariate analysis

Initially, we performed the analysis of canonical variables to verify the interrelationship between the variables evaluated (noise and dusts) and the postharvest pre-processing and storage conditions of unloading, cleaning and expediting (Table S1). This technique is similar to main components, but it allows considering the residual variation between repetitions of the same treatment (processing condition in this case).

Posteriorly, we estimated Pearson's correlation coefficients to verify the association between variables in processing conditions. We used the correlation network to graphically express the results. In this procedure, green lines link variables with positive correlation and red lines join negatively correlated variables. The line thickness is proportional to the magnitude of the correlation.

### Algorithm to manage occupational safety

For the occupational health and safety management, a flexible tool must be applied that uses logical methods, in order to adapt to different types of activities. For this, are fundamental the identification and assessment of occupational hazards and safety standards and, in complement, the establishment of actions and their follow-up^44^.

From this, it becomes important to apply a method of assessing the level of occupational safety regulation compliance for grain storage facilities, identifying the regulatory requirements in conformity and non-conformity, in order to ensure the safety management and the standards compliance.

Based in Zhang et al.^45^ study was developed the concept of the Algorithm to manage occupational safety at grain handling and storage facilities. The first step was to identify the hazards by operation (Table 1) and define safety standards and regulations applied directly to grain handling and storage facilities (Table 2).

**Table 1:** Occupational hazards at grain handling and storage facilities by process and activities.

Process	Activity	Noise	Heat	Dust	Confined space	Biological	Ergonomic	Grain entrapment	Machine entanglement	Fire and Explosion	Falls
Receiving	Manual sampling	X	–	–	–	X	X	–	–	–	X
	Automated sampling	–	–	–	–	–	X	–	–	–	–
	Weighing the load and vehicle	–	–	–	–	–	X	–	–	–	–
	Vehicle and load information registration of information and input documentation generation	–	–	–	–	–	X	–	–	–	–
Unloading	Automated pneumatic lift unload	X	–	X	–	–	–	–	X	–	–
	Gravity unload	X	–	X	–	–	X	–	–	–	X
	Hoppers product flow	X	–	X	X	X	–	X	X	X	–
Pre–cleaning	Tunnels for horizontal product transport from hopper to pre-cleaning machines	–	–	X	X	X	–	–	X	X	–
	Pre cleaning machines	X	–	X	–	–	X	–	X	X	X
Drying	Elevators to transport the product from pre-cleaning to the dryer	X	–	X	X	–	–	–	X	X	X
	Tunnels for horizontal product transport from pre-cleaning to dryer	–	–	X	X	X	–	–	X	X	–
	Furnace	–	X	–	–	–	X	–	–	X	–
	Grain dryer	X	X	–	X	–	–	–	X	X	–
	Drying sampling	X	X	–	X	–	–	–	X	X	–
Cleaning	Tunnels for horizontal product transport from the dryer to cleaning machines or to storage silo	–	–	X	X	X	–	–	X	X	–
	Cleaning machines	X	–	X	–	–	X	–	X	X	X
Storage	Tunnels for horizontal product transport from dryer to storage silo	–	–	X	X	X	–	–	X	X	–
	Monitoring stored grains	–	–	–	X	X	–	X	X	X	X
	Sampling stored grains	–	–	–	X	X	–	X	X	X	X
	Purge	–	–	–	X	X	–	X	X	X	X
	Transporting the product from the silo to the discharge point	X	–	X	–	–	–	–	X	X	–
Expedition	Discharge the product in the vehicle	X	–	X	–	X	X	–	–	X	X
	Weighing the load and vehicle	–	–	–	–	–	X	–	–	–	–
	Vehicle and load information registration of information and output documentation generation	–	–	–	–	–	X	–	–	–	–

**Table 2:** Safety standards used in the Algorithm development.

Safety standard	Title	Publication date	Last up date
NR 31	Occupational safety and health in agriculture, livestock, forestry, forestry and aquaculture	March 2005	December 2018
NR 33	Safety and health in work in confined spaces	December 2006	July 2019
NR 35	Work at height	March 2012	July 2019
RT n.° 22	Silos and warehouses	April 2017	–

**Figure 3:**
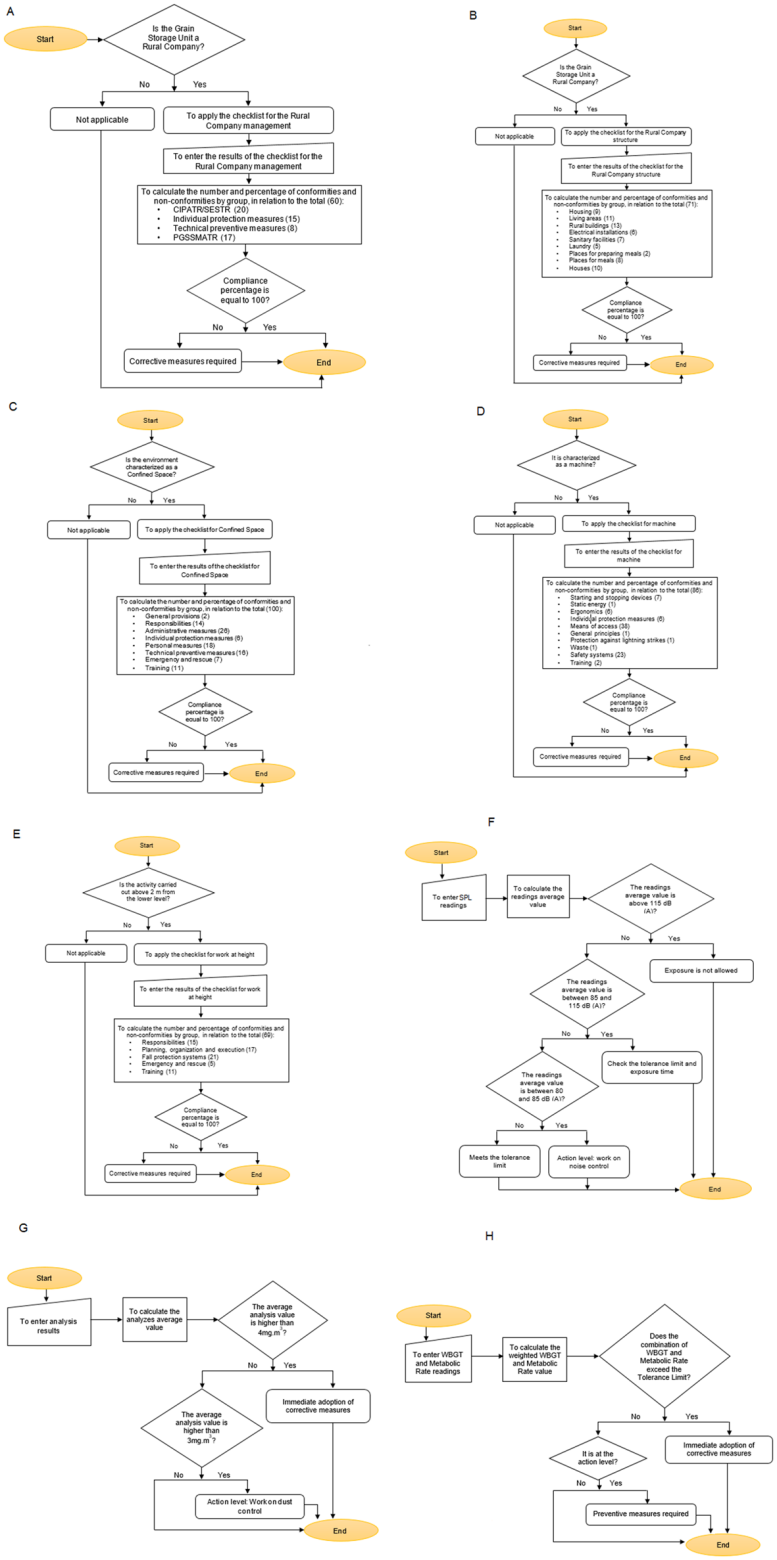
Chart flow to algorithm development on rural company safety management (**A**), chart flow to algorithm development on rural company safety structure (**B**), chart flow to algorithm development on safety at confined space (**C**), chart flow to algorithm development on safety at machinery and equipment (**D**), chart flow to algorithm development on safety at work at height (**E**), chart flow to algorithm development on noise measurement (**F**), chart flow to algorithm development on dust measurement (**G**), chart flow to algorithm development on heat stress measurement (**H**).

The concept of the algorithm was developed based on the hazards at activities and processes at grain handling and storage facility, and in the applicable safety standards. In that way, was composed the flowcharts for the rural company management, rural company structure, confined space, machinery and equipment, work at height and quantifiable occupational hazards of noise, dust and heat (Fig. 3A–H).

Finally, the concept of algorithm comprises four screens set, starting to register screens, verification screens, reports screens and action follow-up screens. The register screens are to register users, the grain handling and storage facility, the facility processes, the process machinery and equipment and the selected safety standards.

**Figure 4:**
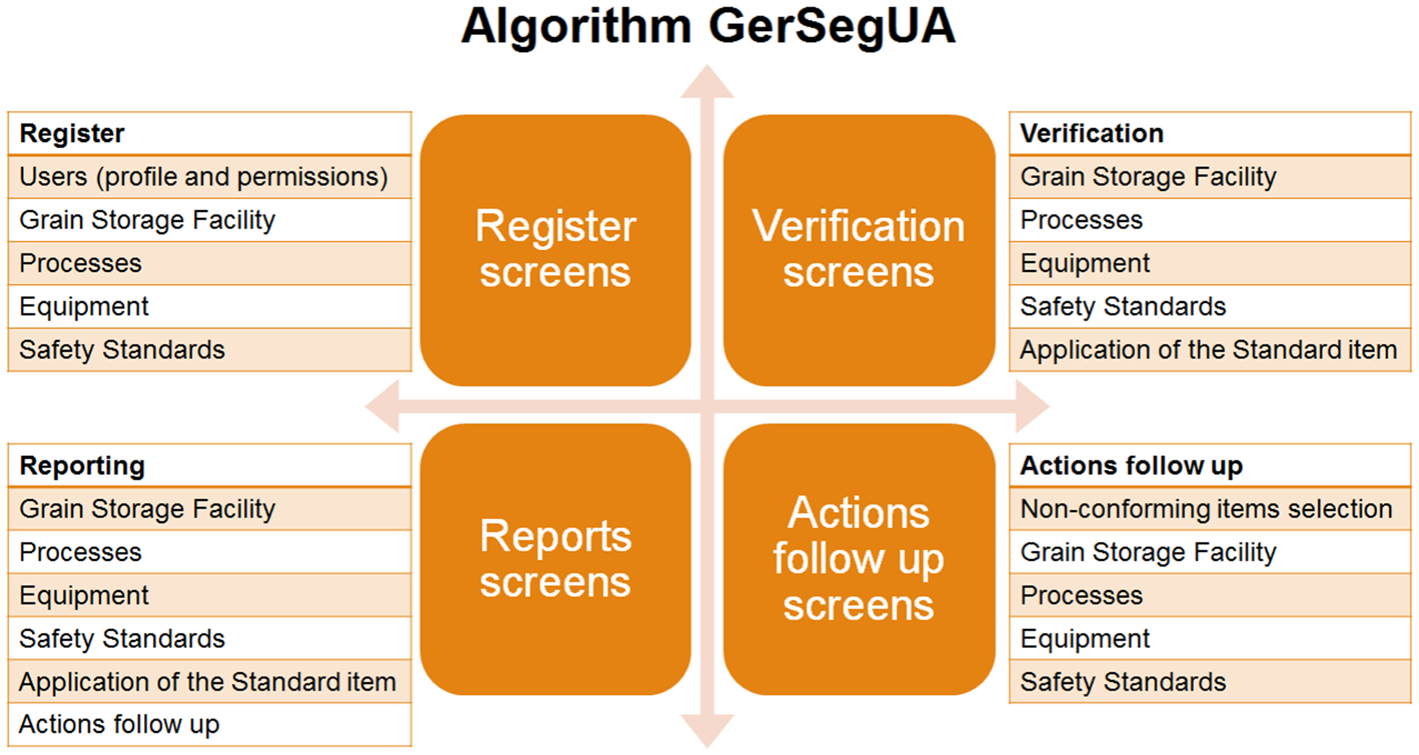
Algorithm development screens set.

The verification screens are to verify the conformity with safety standards, the verification could be by grain handling and storage facility, process, machinery and equipment, standard or by standard item (Fig. 4).

The reporting screens are to report the items compliant and non-compliant and the compliance percentage by grain handling and storage facility, process, machinery and equipment, standard or by standard item. Besides that, there are reports related to actions follow up.

The actions follow-up screens are to assign actions to solve non-compliant items, with action description, why the action is necessary, where action is going to be implemented, responsible person, target dates, how much should be the investment ad how will be implemented.

## Results and discussion

### Noise

**Figure 5:**
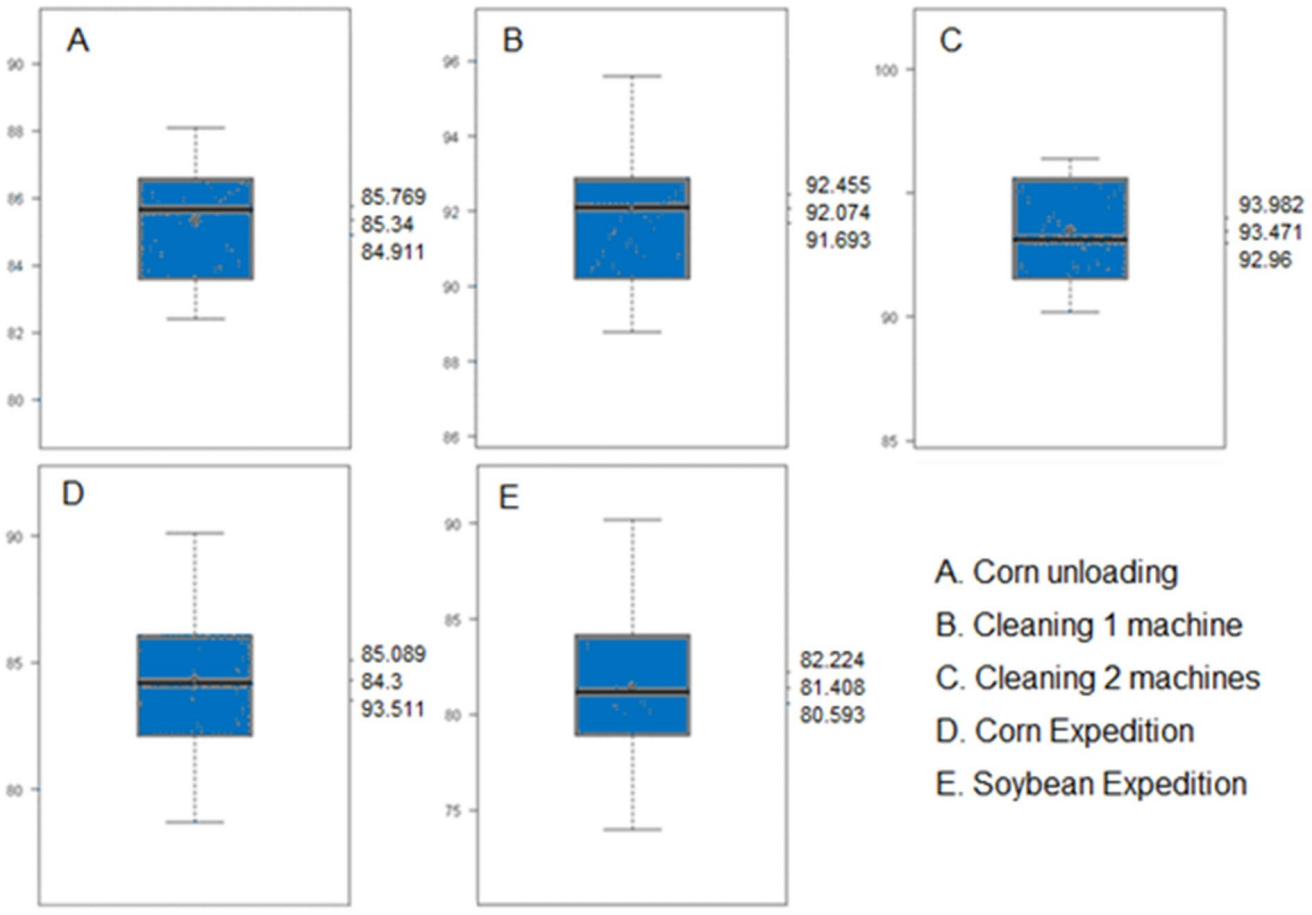
Noise level measurement on Upper Confidence Limit (UCL), average and Lower Confidence Limit (LCL) at corn unloading (**A**), corn cleaning with one machine (**B**) operation, corn cleaning with two machines (**C**) operation, corn expedition (**D**) and soybean expedition (**E**).

Occupational noise is present in agricultural activities, with an emphasis on pre-processing and storage postharvest operations^46^. The pre-processing and storage operations as grains unloading, cleaning machinery and shipping operation have prevalence of occupational noise at grain storage unit^9^. The noise at these operations was quantified and the results are showed at Table 3 and Fig. 5.

**Table 3:** Noise level measurement results at grain unloading (U), cleaning with one machine (C1) operation and two machines operation (C2), and expedition (E) postharvest operations.

Noise level dB (A)													
		Minimum		Average		Maximum		Std deviation		LCL		UCL	
Task	Sample	Soybean	Corn	Soybean	Corn	Soybean	Corn	Soybean	Corn	Soybean	Corn	Soybean	Corn
U	I1	–	84.6	–	86.5	–	88.1	–	1.069	–	86.1	–	86.9
U	I2	–	83.1	–	85.2	–	87.3	–	1.73	–	84.5	–	85.8
U	I3	–	82.4	–	84.3	–	86.5	–	1.51	–	83.7	–	84.9
U	All	–	82.4	–	85.3	–	88.1	–	1.695	–	85	–	85.7
C1	P6	–	91.9	–	94.2	–	95.6	–	1.457	–	93.7	–	94.7
C1	P3	–	88.8	–	90.6	–	92.1	–	1.357	–	90.2	–	91.1
C1	P1	–	89.1	–	90.9	–	92.5	–	1.135	–	90.5	–	91.3
C1	P5	–	91.2	–	92.6	–	93.5	–	0.606	–	92.4	–	92.8
C1	All	–	88.8	–	92.1	–	95.6	–	1.862	–	91.8	–	92.4
C2	P6	–	94.4	–	94.8	–	95	–	0.199	–	94.2	–	95.4
C2	P7	–	95.3	–	95.9	–	96.4	–	0.364	–	94.8	–	97
C2	P5	–	95.6	–	95.9	–	96.2	–	0.2	–	95.3	–	96.5
C2	P3	–	91.2	–	92.3	–	93.6	–	0.851	–	89.7	–	94.8
C2	P4	–	91	–	92.6	–	93.6	–	1.03	–	89.5	–	95.6
C2	P1	–	90.2	–	90.8	–	91.3	–	0.414	–	89.5	–	92
C2	P2	–	90.5	–	92.2	–	93	–	0.885	–	89.5	–	94.8
C2	All	–	90.2	–	93.5	–	96.4	–	2.002	–	93	–	94
E	EV	77.3	86	82.8	86.6	90.2	87	3.695	0.551	81.4	85.7	84.2	87.6
E	E1	80.2	81.9	83.3	86.5	89.4	90.1	2.555	2.495	82.3	85.4	84.3	87.6
E	E2	76.9	82.1	80.2	84.6	87.6	86.1	3.159	1.27	79	84	81.4	85.1
E	E3	74	78.7	78	81.5	84.9	82.9	3.354	1.281	76.7	81	79.2	82.1
E	All	74	78.7	81.5	84.4	90.2	82.9	3.765	2.685	80.8	83.7	82.2	85

Where: LCL = Lower Confidence Limit; UCL = Upper Confidence Limit.

The average sound pressure level, generated in the receiving process, during the corn grains unloading from the transport vehicle to the hopper, using the pneumatic truck dumper, was 85.3 dB (A), with a standard deviation of 1.695. Of the three unloading points evaluated, the beginning of corn grains unloading (I1) obtained the highest noise result. The statistical analysis showed that the lower confidence limit was higher than the tolerance limit of 85 dB (A), thus, there is a 95% probability that the exposure at the beginning of the grains unloading is non-compliant.

To avoid noise-induced hearing loss, the occupational noise exposure should be limited, relating the amount of time to the intensity of noise, during the working day and tolerance limits were established^47^. According to NR 15, the maximum allowable daily exposure in an eight-hour working day is 85 dB (A), and the exposure should be reduced as the sound pressure level increases.

In contrast, the measurements taken in the middle and at the end of the discharge vehicle generated an upper confidence limit below the tolerance limit, which leads to a 95% probability that this is an exposure in accordance with the tolerance limit of 85 dB (A), but above the action level, established by the Brazilian Regulatory Standard NR 9, of 80 dB (A). In this case, it is necessary to adopt noise monitoring and control measures.

From the quantified noise data, the noise dose to which the worker is exposed when carrying out the activity of corn grains unloading was calculated, during the entire working day journey. For this, the work cycle was considered, which includes the tasks of: (a) waiting for the vehicle to enter the hopper area; (b) the vehicle's entrance; (c) making adjustments, such as opening and closing the truck body, raising and lowering the truck load cover, among others; (d) unloading the grains; and, (e) the vehicle exit.

For the calculation of the noise dose, were considered the time in each task, the noise level and the respective tolerance limit. In the daily work journey, 80 work cycles are possible, in which case the calculated noise dose is 0.79 and the equivalent noise level (TWA) is 84 dB (A), a value that does not exceed the tolerance limit, but it reaches the action level given by NR 9, of 80 dB (A), in which it is necessary to adopt risk monitoring and control measures.

The technologies application on postharvest operations can reduce the operator hazards exposure. At grain receiving process, the use of hydraulic lifts to grain unload from vehicle and main bucker elevator with high capacity is going to increase the vehicles flow, demanding a minimum workforce^48^.

In the case of corn grains cleaning, the results of quantified sound pressure level when only one machine is activated (C1), showed an average value of 92.1 dB (A) and standard deviation of 1.862. The calculation of the lower confidence limit indicated a result higher than the tolerance limit, pointing out, with a 95% probability, that the exposure to noise is non-compliant.

The individualized results of the noise quantification on the operator platform, in front of the machine and on its right and left sides also indicated average values above 90 dB (A), with the highest noise value on the operator platform.

To calculate the noise dose, it was considered the time that the worker spends during the daily work day journey, performing the tasks related to the operation of a cleaning machine, in each position, in which the noise was evaluated, in addition to other tasks that the operator can do. The calculated noise dose is 2.17 and the equivalent noise level (TWA) is 88.4 dB (A), a value that exceeds the tolerance limit.

In addition, the two cleaning machines working together noise level (C2) was quantified. In this case, the average noise was 93.5 dB (A) and the standard deviation was 2. This result characterizes an exposure above the tolerance limit, with a 95% confidence level. The noise dose calculated for the exposure during the entire working day in the operation of the two machines is 3.19 and the equivalent noise level (TWA) is 90 dB (A).

When the action level is exceeded, noise attenuation measures at its source or in its trajectory are recommended. As an alternative, while noise mitigation measures are implemented, the operator time of exposure to the hazard could be reduced or limited, alternating the operation of cleaning machines with the execution of other activities in an environment with the noise level below the noise tolerance limit.

This result corroborates the fact that, in the grain storage unit, the cleaning machines emit occupational noise above the tolerance limit^49^, as well as the machines in the seed processing unit^41^.

The expedition operation (E) was quantified in relation to noise for corn and soybean grains. In the corn grains expedition, the values quantified at points E1, E2 and E3 showed increasing as they approach the additional noise source, constituted by the vehicle's engine. The task of entering the vehicle in the expedition area and the point located in the front of the vehicle showed values above the tolerance limit, with a 95% confidence level.

In addition, the noise dose to which the operator is exposed was calculated when carrying out the activity of corn grains expedition during the entire working day journey, considering the time spend in each task, the noise level and the respective tolerance limit.

For this, the work cycle was considered, which includes the tasks of: (a) waiting for the vehicle to enter the dispatch area; (b) the vehicle's entrance; (c) making adjustments, such as closing the openings in the vehicle body, adjusting the vehicle, among others; (d) filling the vehicle body with grains; and, (e) leaving the vehicle from the expedition area. In the daily workday 41 work cycles are possible and, in this case, the calculated noise dose is 0.79 and the equivalent noise level (TWA) is 84 dB (A), a value that does not exceed the limit of tolerance, but reaches the action level given by NR 9, and it is necessary to adopt risk monitoring and control measures.

The soybean grains expedition operation showed lower noise values at the three measurement points, compared to the corn grains expedition. However, for both corn and soybean, the highest values were found at the point located at the front of the vehicle, closest to the engine, characterized as an additional source of noise. These data are corroborated by Tzivian et al.^50^ when they emphasize that the flow of vehicles influences the noise level to which employees are exposed. In this sense, it is worth mentioning that the vehicle remains activated throughout the grain loading period.

The occupational exposure to noise in the soybean grains expedition process was characterized as complying with the legislation, with 95% confidence, since, in all points, the upper limit of confidence was lower than the limit of tolerance. The noise dose calculated for the soybean grains expedition process is 0.59 and the TWA is 82.7 dB (A), with 49 work cycles being possible during the daily shift. Occupational noise is characterized by any undesirable, unpleasant and disturbing sound^29^. The prolonged and continued noise exposition can cause a chronic and irreversible disease called noise-induced hearing loss^51^. Multiple physical occupational hazards exposures are related to the increase of the risk of hearing loss^52^.

Preventive measures to control and attenuate noise in the generating source, in its trajectory and, finally, individual protections are recommended. According to Firth et al.^53^ noise appears as an occupational hazard in the agricultural sector, with about one third of workers exposed daily to levels above 85 dB (A). In addition, operators who make use of hearing protection equipment are rare^54^. In carrying out this study in the storage unit, it can be seen that there is no established hearing conservation program, and the use of hearing protectors has not been evidenced.

Measures to mitigate occupational noise at the source or in the path include acoustic insulation of walls and/or ceilings, redistribution of the arrangement of machines and equipment in the environment, as well as their concentration and, finally, the enclosure of the machine. Enclosure can be carried out by means of booths or acoustic barriers, noise attenuators and acoustic doors. Silencers can also be used to prevent airborne noise from being dissipative or resonant. Another very important point is the proper maintenance with repairs and replacement of parts that are causing the noise, finally to replacing the machine with a soundless one.

Gasques et al.^49^, assessing occupational noise in a grain storage unit found exposures above the tolerance limits. However, they found that the use of personal protective equipment mitigates the risk. This fact demonstrates the importance of adequated personal protective equipment and its correct use, because to recommend the wearing of PPE does not always mean effective protection^55^^)^. In addition, potential strategies for hazards control at grains storage units could be related to increase the operator level of safety and health education^54^^)^.

### Noise map

The noise maps were created for the area of 30 m long by 15 m wide, in which the grain unloading in the hopper and the respective grain cleaning occurs. Oliveira et al.^41^ detailed the existing noise in the workplaces evaluated in their work through the use of noise maps, which are efficient in determining hazard zones and serve to make decisions, aiming at the operator safety and health.

**Figure 6:**
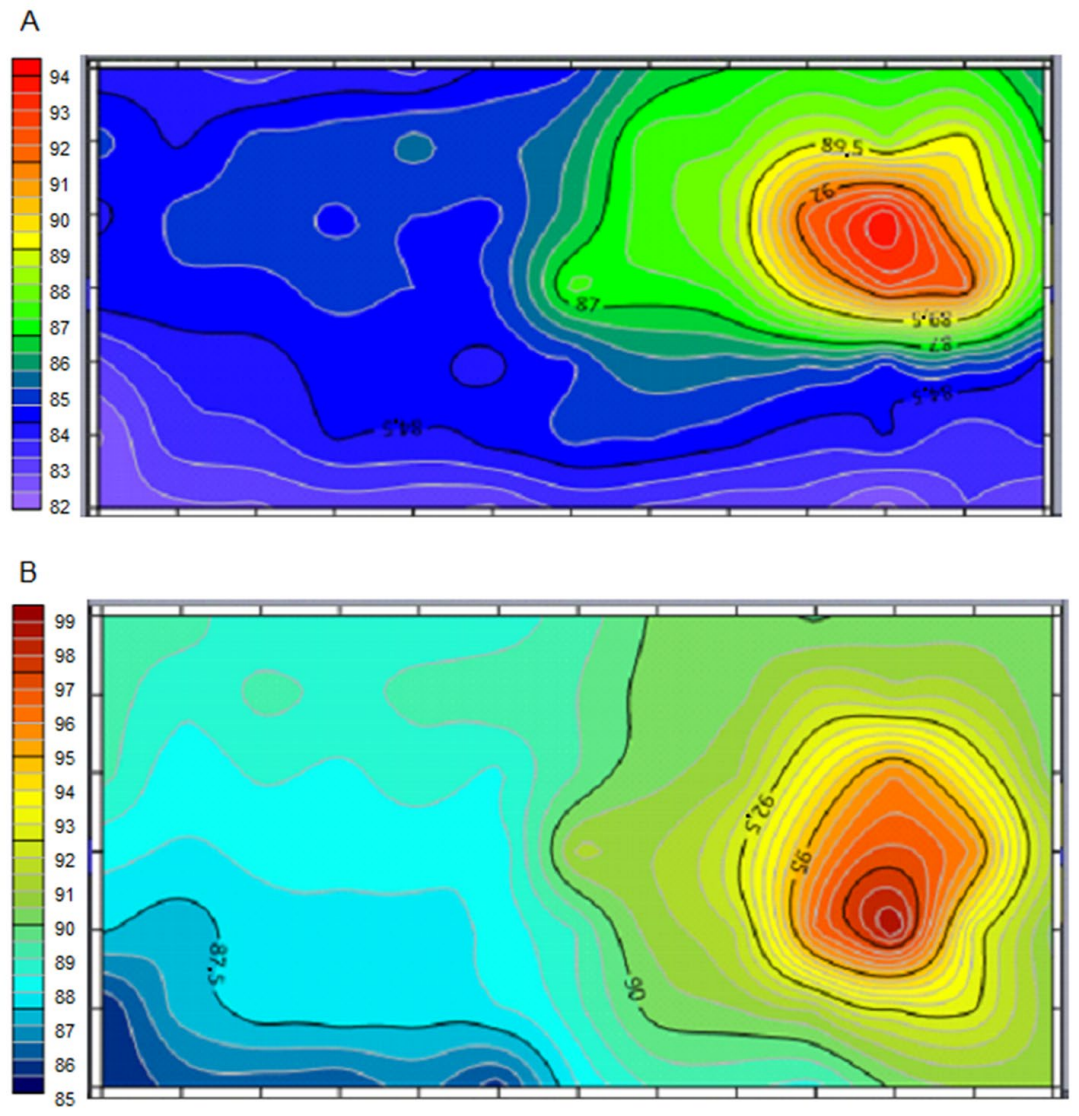
Noise level map measurement results at grain unloading and cleaning area with one machine (**A**) operation and two machines operation (**B**).

The first map was created with only one grains cleaning machine activated (Fig. 6A), in which the maximum value was 94.4 dB (A). The blue curves represent the areas in which the tolerance limit has not been exceeded. However, analyzing the environment, there are no places with values below the control level of 80 dB (A), the lowest value being 82.3 dB (A).

The central area of the place, on the green curve, of 87 dB (A), comprises the location of the grain elevator, which is activated together with the cleaning process, in order to promote the transport of the product to the cleaning machines, this equipment is an additional source of noise.

Figure 6B was constituted with the two grains cleaning machines, installed side by side, and operated together. The noise readings are higher than 85 dB (A) and reach 99.1 dB (A), proving to be an unsafe environment in all sampled points. Readings above 90 dB (A) advance to the vicinity of half the building, including the unloading area and the grain elevator area.

Any activity that may be carried out in the grain receiving area, when the cleaning machines are activated, must be evaluated in terms of individual protection and exposure time, due to the high level of sound pressure verified. It is important to note that continuous and prolonged exposure to noise causes irreversible hearing loss to workers^29^.

This configuration of equipment disposition is common to be found in the storage units, with the unloading of grains, hoppers and cleaning machines located in the same environment, with the use of grain elevators to transport them from one operation to the other.

### Dust

Dust is generated by the mechanical solid rupture^42,56^ and constitutes a hazard in agricultural activities. The grain dust exposure also occurs in the post-harvest operations and consists on particles of organic and inorganic materials, such as soil elements, plant fragments, rodent excrement, mite and insect parts, pesticides residues, fungi, bacteria and mycotoxins^27,57^.

**Figure 7:**
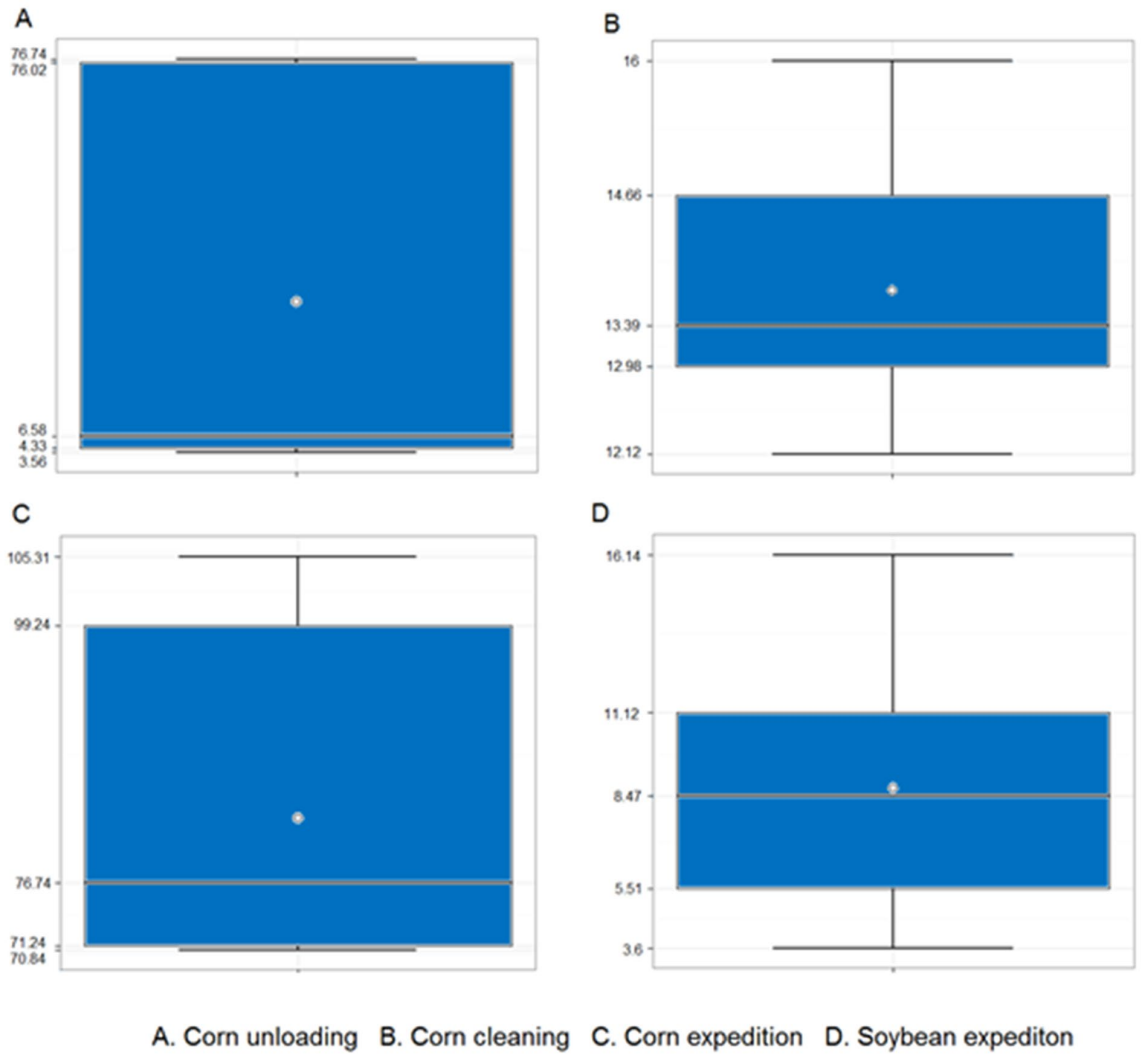
Total dust measurement on Upper Confidence Limit (UCL), average and Lower Confidence Limit (LCL) at corn unloading (**A**), corn cleaning (**B**) operation, corn expedition (**C**) and soybean expedition (**D**).

The dust quantification in the environment was carried out in the pre-processing and storage operations of corn unloading in the hopper, corn cleaning and corn and soybean expedition, the number of samples, statistical analysis with the average values, Lower Confidence Limit (LCL) Upper Confidence Limit (UCL), and operator workday exposition probability are presented at Table 4 and Figs. 7A–D.

**Table 4:** Total dust measurement at unloading (U), cleaning (C1 one machine; C2 two machines) and expedition (E) postharvest pre-processing operations.

Dust concentration																
Task	n		Sum Y2		Average Y		Std deviation		Tabulated value		Probability		LCL		UCL	
	Soybean	Corn	Soybean	Corn	Soybean	Corn	Soybean	Corn	Soybean	Corn	Soybean	Corn	Soybean	Corn	Soybean	Corn
Unloading	–	8	–	5.506	–	0.629	–	0.032	–	19.71	–	100	–	8.11	–	8.13
Cleaning	–	7	–	1.856	–	0.511	–	0.492	–	1.04	–	85.08	–	3.26	–	3.29
Expedition	7	5	0.755	8.494	0.252	1.302	0.169	0.985	1.49	1.32	93.19	90.66	1.99	20.24	2.02	20.27

Where: LCL = Lower Confidence Limit; UCL = Upper Confidence Limit.

The corn grains unloading process showed dust quantified values above the tolerance limit, with the Lower Confidence Limit (LIC) value greater than 1, confirming the hypothesis of non-compliance, in which the tolerance limit is exceeded with 95% certainty. In addition, after carrying out the statistical treatment of the data, it was calculated that the probability of the dust exposure in the corn unloading process is 100% of the workdays.

The result of non-compliance was also observed in the corn grains cleaning process, and from the statistical treatment of the data, there is a probability of the dust exposure in 85.08% of the workdays. The value of the LIC is greater than 1, confirming the hypothesis of non-compliance, in which the tolerance limit is exceeded with 95% certainty.

Regarding to corn grains expedition process, the dust values obtained, show the LIC is greater than 1, confirming the hypothesis of non-compliance, with the tolerance limit being exceeded with 95% certainty. Through the statistical treatment of the data, it was found that the probability of the dust exposure is 90.66% of the workdays.

Regarding soybean grains, the dust concentration generated during the expedition process presented results were the concentrations were lower than those quantified in the corn expedition, but the concentration exceeded the tolerance limit. The value of the LIC is greater than 1, confirming the hypothesis of non-compliance, in which the tolerance limit is exceeded with 95% certainty. Nevertheless, when the data are processed statistically, the probability of dust exposure obtained in the soybean grains expedition process is 93.19% of the workdays.

Dust measurement studies at postharvest operations have been carried out in Canada, Britsh Columbia^25^; United States, Ohio^58^; Brazil, Rio Grande do Sul^41^ and Costa Rica^20^. Chan-Yeung et al.^25^ performed dust measurements on Canadian grain elevators terminal in 14 years’ period and saw dust concentrations decrease from total dust of 10.1 mg m^-3^ in 1974 to 1.9 mg m^-3^ in 1988, what shows the efficiency of hazard control measurements. Regarding the operation, the soybean unloading and handling had higher dust particles concentration compared with soybean harvesting in an Ohio farm^58^.

A soybean processing plant in southern of Brazil has an elevated total dust concentration^41^, in that way, Rodrigues-Zamora et al.^20^ found that operators of grain storage facilities in Costa Rica are exposed to elevated inhalable dust concentrations, mostly above international exposure limits as well on the Iowa farms postharvest operations^27^. High dust concentration was also observed at almonds, melon and tomato crops^27^. The cleaning activity in small spaces and use of compressed air increases the likelihood of dust concentrations^27^.

Combining the method of questionnaire, examinations and measurement, Chan-Yeung et al.^25^ confirmed that grain dust has adverse effects on the lungs and grain workers still had lower lung function compared with other workers. Exposure to grain dust accentuated respiratory disorders with declines in lung functions^59^. Lung dust deposition occurs due dust particles in high concentration in the environment and the exposure time longer than the organism has to be able to eliminate them^58,60^. The quantitative particulates evaluation, through workplace studies, combined with the adoption of control measures, contribute to avoid harmful health exposure^26,56^.

In this context of results, it is important to observe the implementation of control measures and effective immediate individual protection measures, as there was no constancy in the use of respirators during the execution of the activities of the evaluated processes, as well as in the study by Sauvé et al.^27^. In contrast, on Geng and Jepsen^54^ study, more than 90% of the Ohio farmers reported using any kind of respiratory protection during grain handling work.

Regarding the dust, engineering and control measures are necessary to minimize risk in the workplace^42^. Among the engineering measures, it is possible to mention the application of process confinement, process segregation, operation dust extraction using a dust collecting system with external or internal booth, exhaust systems and diluting ventilation^42^.

In addition, it is fundamental an efficient facility housekeeping procedure and application^61^. Teixeira et al.^62^ showed the efficiency of using the exhaust system in the reduction of dust concentration in furniture factories, highlighting the importance of system efficiency and sizing, as well system maintenance, with frequent residues discharge.

Measures related to workers concern the limitation of exposure time, education, training, personal protective equipment and medical control^42^^)^. Potential strategies for agricultural dust hazard control could be related to increase the operator level of safety and health education^63^.

### Heat

The thermal stress quantification, by the Wet Bulb Globe Thermometer Index (WBGT), considered the exposure cycle in the wood supply to the furnace, in the period of 60 consecutive minutes.

The activities performed by the worker correspond to supplying the furnace, followed by a period of rest and rehydration; grains sampling in the grains drying process, both in the same environment where the furnace is located; and, to get and to bring the firewood in a wheel hand cart to the side of the furnace from an external environment, with solar charge. The main results point to a WBGT with a value of 43.64 and a metabolic rate of 315 in the task of filling the furnace (Table 5).

**Table 5:** Thermal stress measurement at wood supply to the furnace activity.

Task	Time (min)	Task classification	M (W)	T(°C)	Tw(°C)	Tg (°C)	WBGT (°C)	WBGT* (°C)
Feed the furnace	5.5	Heavy work with both arms	315	44.5	26.2	50.6	43.64	–
Rest	31	Seated	100	28.4	22	29	29.9	–
Grains sampling	12.5	Light work with both arms	243	28.4	29	29.9	29.9	–
To get the firewood	9	Moderate lifting or pushing work	349	25.8	25.2	23.23	–	23.23
To bring the firewood in a wheel cart	2	Work of pushing wheel cart, on the same plane, with load	391	25.8	25.2	23.23	–	23.23

Where: M = metabolic rate; WBGT * = outdoor activities with direct solar charge.

Thus, the calculation of the metabolic rate weighted average, relative to the physical activities performed by the worker, shows a value of 197 W. The value of the WBGT weighted average, in the same 60 min considered in the calculation of the metabolic rate, is 29.9 °C. Considering the limits established in NR 15, this occupational exposure does not exceed the tolerance limit, which is up to 30.3 °C of WBGT for a metabolic rate of 197 W.

The compliance of the activity with the tolerance limit differs from the result found by Monteiro et al.^64^. These first evaluated the thermal stress in a furnace firewood supply activity at a biscuit industry, while Monteiro et al.^64^ evaluated in boiler furnace firewood feeding, in which the operator monitors the operation of the equipment, seated in a place close to it. Such fact may have been the differential in the thermal overload presented in the activities performance.

The occupational heat exposure can cause some diseases, as the heat syncope, dehydration edema, cramps and hyperthermia^39^. A factor that contributes to heat diseases are air temperature, radiant temperature, humidity, air speed, the clothes used and the activities performed^65^. Occupational heat stress is also associated with injuries due the physical discomfort, fatigue and loss of concentration^21^.

To prevent the heat exposure consequences, action as an intermittent work regimen with rest periods, intake of liquids and salts, installation of fan, extractor, thermal barriers, training, as well as the use of personal protective equipment are preventive measures that should be adopted^21^.

### Statistical multivariate analysis

**Figure 8:**
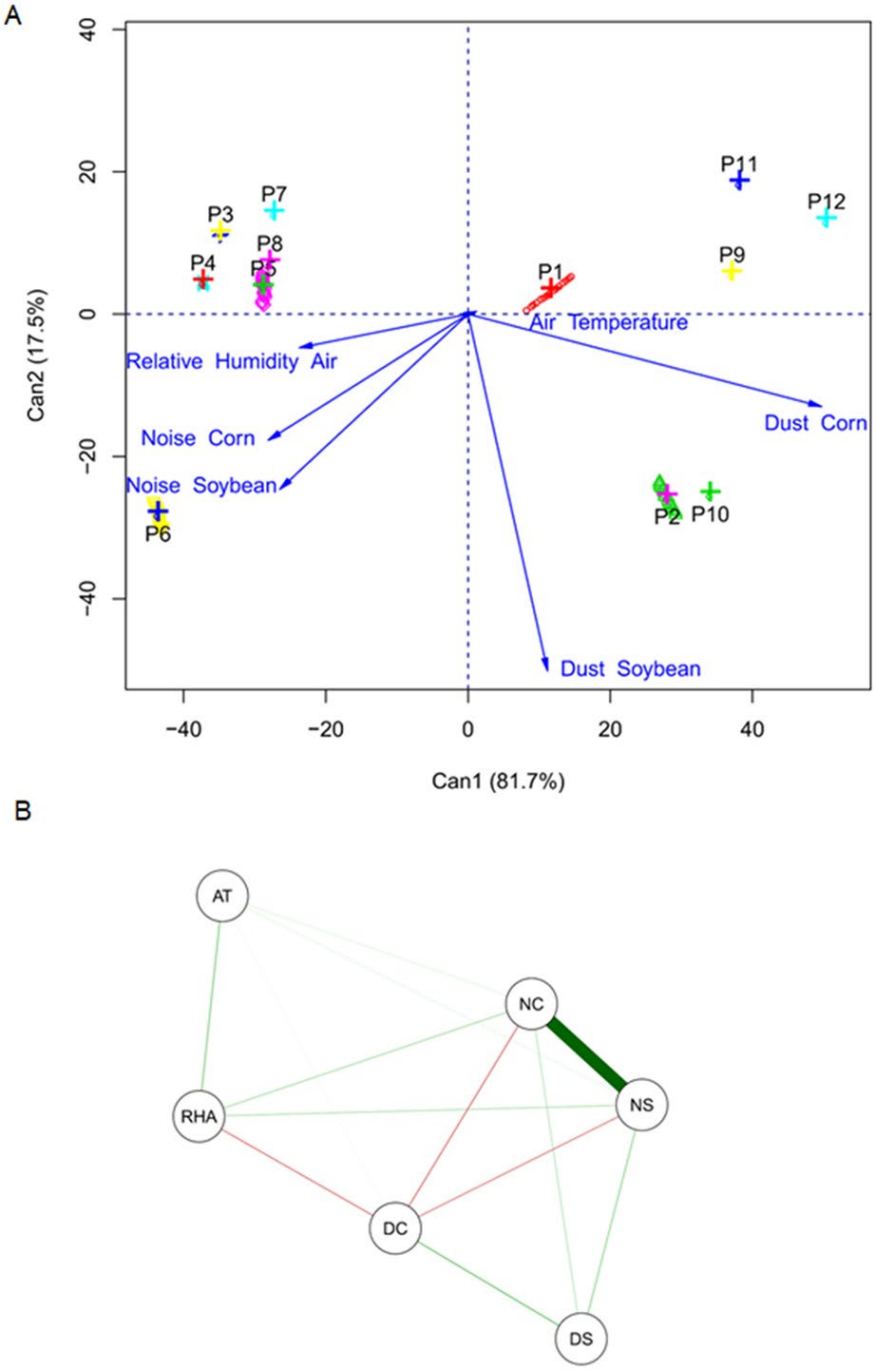
Canonical variables (**A**) for noise in corn (NC), noise in soybean (NS), dust in corn (DC), dust in soybean (DS), relative air humidity (RHA) and air temperature (AT) evaluated in different processing conditions (P). Person's correlation network (**B**) between the variables noise in corn (NC), noise in soybean (NS), dust in corn (DC), dust in soybean (DS), relative air humidity (RHA) and air temperature (AT) evaluated in different processing conditions.

The analysis of canonical variables gathered in the biplot presented 99.2% of the total variation between treatments for the evaluated variables. This result demonstrates high credibility in the interpretation of the following results. Thus, treatments (postharvest pre-processing and storage operations) that are close in the Fig. 8A have high similarity. The vectors (arrows) point to the variables that most influenced the similarity of specific treatments.

Clusters P3, P4, P5, P7 and P8 were similar, with the same behavior especially with regard to relative air humidity, whose highest values were obtained in grain unloading and cleaning pre-processing operations. The grain cleaning condition P6 stood out among the others for obtaining the highest values of noise for corn and soybean. P1 stood out for air temperature.

It is possible to observe 4 distinct groups with different behaviors. Clusters P3, P4, P5, P7 and P8 were similar, with the same behavior, without influence of the analyzed variables, except the relative air humidity. The same occurred with the clusters P1, P9, P11 and P12 with little influence of the air temperature and the lowest noise levels, which correspond to the grain unloading and grains expedition operations. Clusters P2 and P10 stood out for presenting the highest values of dust for corn and soybean. Finally, cluster P6 represents the grains cleaning operation, with the highest values of occupational noise.

It is possible to notice, at clusters P2 and P10, the inversely proportional influence of the relative air humidity in the presence of dust, that is, for lower values of relative humidity, the higher the values relative to the dust concentration in the environment. In addition, the air temperature does not show much influence on the dust concentration. Cluster P6, shows relative air humidity, has a direct influence on the occupational noise level and the air temperature has an inversely proportional influence on the noise level.

In relation to operations, cleaning showed the highest level of occupational noise, on the other hand, the highest concentration of dust is in the operation of corn grains expedition. This fact occurs due to the grain cleaning process mechanization and the simultaneous movement of a larger amount of dry grains during the expedition process, which generates a higher dust concentration.

Thus, the operation that presented the lowest values for occupational noise is grains expedition, but with higher values related to dust concentration. In the grain expedition operation, the height of grains displacement fall is lower, when compared to the operation of grain unloading from the transport vehicle to the hopper. Regarding the operations, the values of total dust in the grain unloading are lower than in the grain expedition, because in unloading operation the grains have moisture content higher than in the expedition operation.

Agricultural workers face multiple occupational hazards exposures and co-exposures at workplace and a multiple exposures seemed to increase the risk of hearing loss^66^. In addition, the ambient air pollution and noise long-term exposition are associated with neurocognitive functions, mood disorders and neurodegenerative disease^50^.

The generated Pearson correlation network is contained in Fig. 8B. The most expressive correlation (r = 0.91 and p-value < 0.05) occurred between noise in corn and noise in soybean in a positive way. The other variables showed low magnitude correlations, whose values were not significantly different from zero by the t-test (*p* value > 0.05).

It is possible to notice an inversely proportional correlation of the relative air humidity and the dust concentration. The air temperature and air humidity may affect the concentration of dust components^27^.

In addition, environmental factors of air temperature and humidity can affect the activities performance. Elevation of the indoor air humidity in the office environment may have positive impact at the perceived indoor air quality and work performance^67–69^.

In other hand, elevated temperature increases the symptoms like dry eyes and respiratory symptoms and low indoor air humidity causes symptoms like dry and tired eyes, which deteriorates the work performance^70^. Ventilation should be integrated with both the indoor air humidity and the room temperature in a strategic to perceive better indoor air quality, health, working performance^70^ besides the dust and noise hazard control.

### Algorithm to manage occupational safety

**Figure 9:**
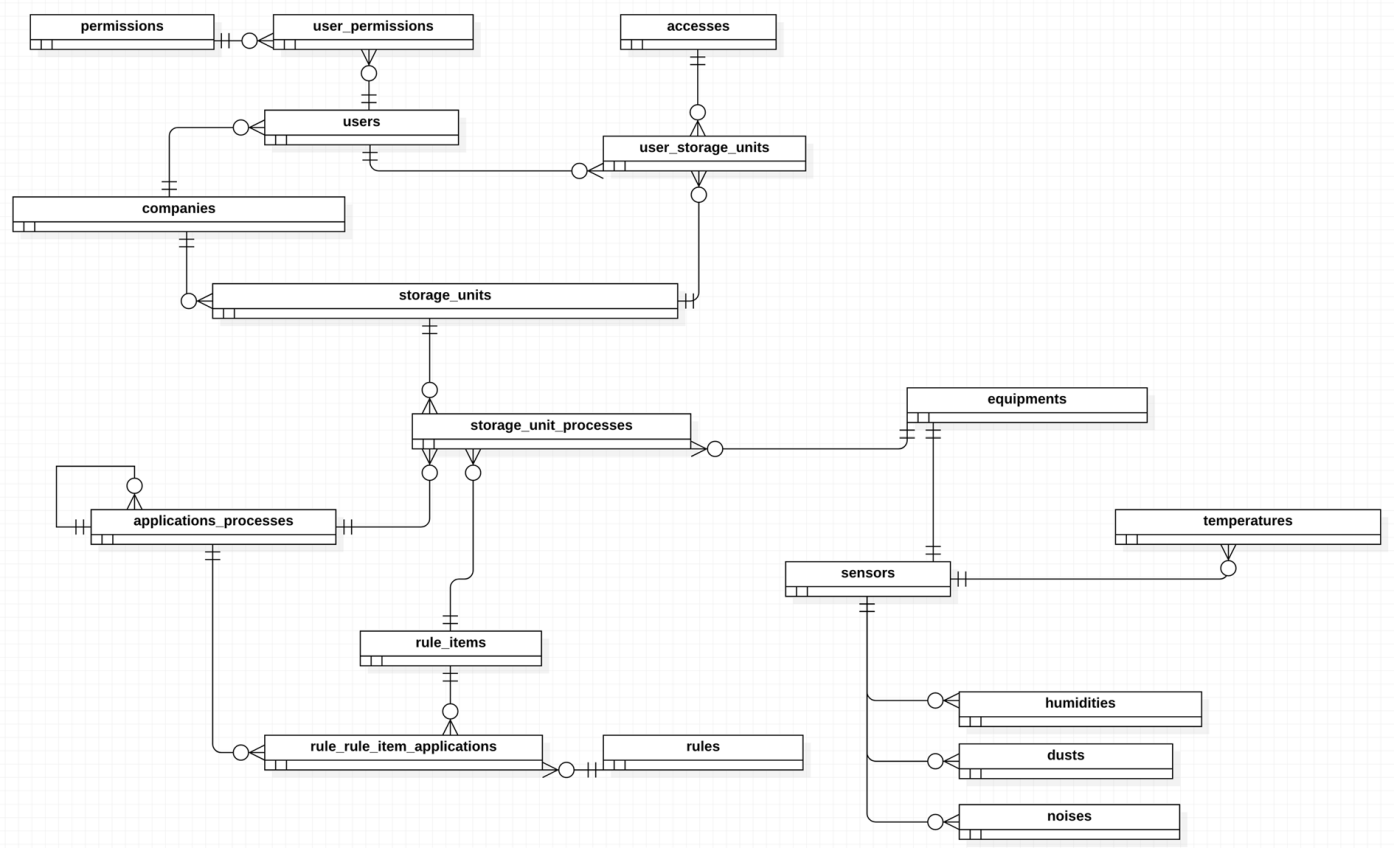
Algorithm simplified to manage occupational safety at grain handling and storage facilities chart flow.

**Figure 10:**
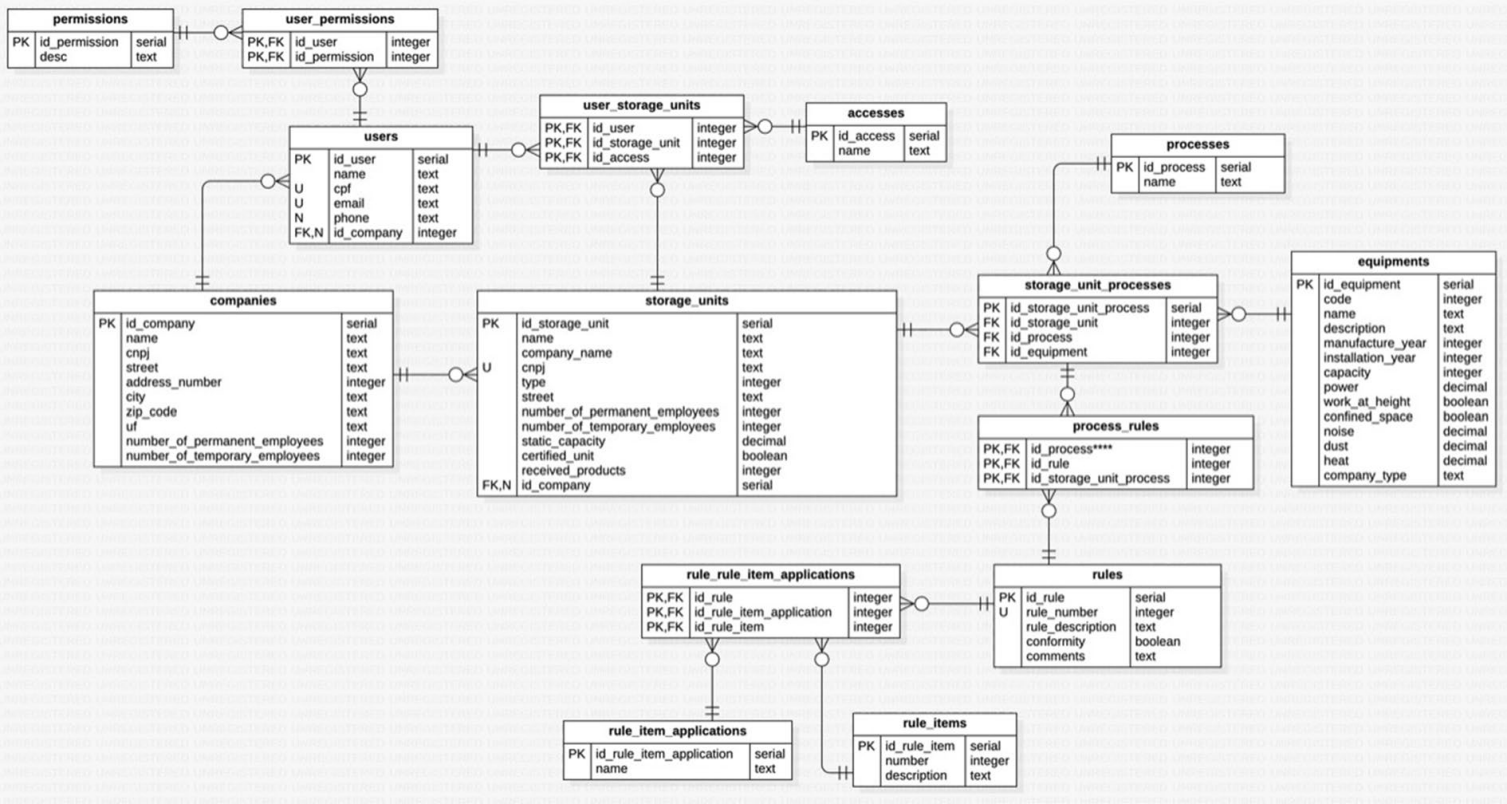
Algorithm detailed to manage occupational safety at grain handling and storage facilities chart flow.

**Figure 11:**
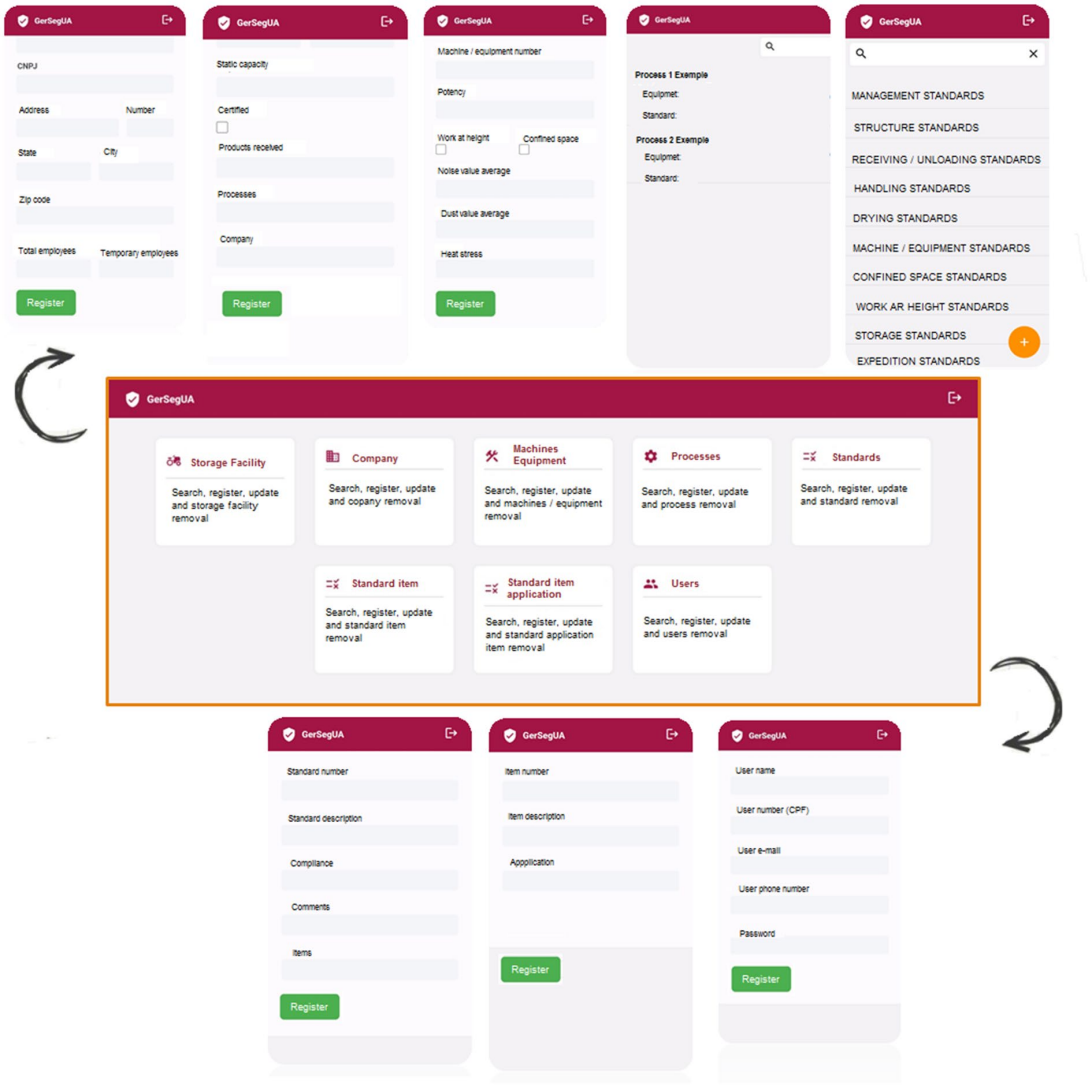
Algorithm to manage occupational safety at grain handling and storage facilities register screens.

Occupational health and safety management is one of the main elements of the companies' management system and is related to quality, standardization and the environment, contributing to operational efficiency^71^. The Algorithm to manage occupational safety at grain handling and storage facilities chart flow in presented in Figs. 9 and 10 the Algorithm register screens in Fig. 11.

Therefore, the ISO 45.001 standard, like OSHAS 18.001, on occupational health and safety management systems, represents an important tool to implement, manage or update occupational health and safety management system^72^. Its structure presents scope, reference norms, terms and definitions, context of the organization, leadership and participation of workers, planning, support, operation, performance evaluation and improvement^73^.

Zhang et al.^45^ developed a quantitative method for assessing the level of occupational safety regulations compliance for companies, identifying items in non-compliance, in order to ensure that safety management complies with the requirements of the standards. In the same way, the proposed Algorithm to manage occupational safety at grain handling and storage facilities assessing the level of occupational safety regulations compliance, in addition to the performance evaluation, it has the possibility to improve the safety compliance by actions assignment and follow up^73–76^.

## Conclusions

Noise and dust were characterized as occupational hazards in the pre-processing and storage operations for corn and soybean grains, and it is important to adopt hazards control and mitigation measures.

The grains cleaning and expedition operations were considered to be the operations that present the higher occupational hazard of noise and dust in grain storage units.

The sampling points determined in the operations of grains unloading, cleaning, drying and expedition characterized the occupational hazards of noise, dust and heat stress present in the grain storage unit.

Noise analysis did not show a difference between grains (corn and soybean), only between operations, being the grains cleaning operation the most critical point.

Dust was the higher occupational hazard observed in the grains storage unit flow. The flow of corn grain mass with low water content, in the pre-processing and storage operations, caused higher dust concentrations in the expedition operation.

In the environment of the pre-processing and storage operations, the relative air humidity variable had a higher influence on the dust concentration and occupational hazard increase.

The heat stress hazard was the higher occupational hazard present in the grain drying operation, by manual supplying the furnace.

It was concluded that the proposed evaluation and the method applied to characterize and quantify the noise, dust and heat stress hazards in grain storage units were satisfactory. Bearing in mind that until today there is no recommendation for a sampling plan that identifies the location of the data collection points and application of occupational hazards assessments for the control and monitoring, in conjunction with the norms and guidelines of the countries or region, this method is recommended as standard, for use in corn and soybean grains handling and storage units.

The Algorithm to manage occupational safety at grain handling and storage facilities is a simple and practical tool to identify and monitor the safety compliance on postharvest operations.

## Supplementary Information

Supplementary Information.

## Data Availability

The experimental research and field studies on plants and plant material were comply with local and national regulations. The study complied with institutional, national, and international guidelines and legislation. The authors complied with the IUCN Policy Statement on Research Involving Species at Risk of Extinction and the Convention on the Trade in Endangered Species of Wild Fauna and Flora for the collection of plant or seed specimens. The authors declare that no wild plants were collected and/or used in this scientific work.
