# Supplementary material for: Towards a software architecture to manage occupational safety at grain handling and storage facilities
Source: Sci Rep. 2022 Feb 16;12:2612. doi: 10.1038/s41598-022-06534-8 (PMC8850590; doi:10.1038/s41598-022-06534-8)
Supplement: Supplementary file 1 — Supplementary Information. [file 41598_2022_6534_MOESM1_ESM.docx]

**Supplementary material**

**Table S1.** Multivariate analysis of canonical variables

| Operations | Grain | Repetitions | Clusters | Noise | | Dust | | Air Relative Humidity (%) | Air  Temperature (°C) |
| --- | --- | --- | --- | --- | --- | --- | --- | --- | --- |
|  |  |  |  | Corn | Soybean | Corn | Soybean |  |  |
| Unloading | Dry | 1 | P1 | 83.1 | 79.1 | 47.43 | 8.08 | 70 | 30 |
| Unloading | Dry | 2 | P1 | 83.1 | 79.1 | 47.63 | 7.98 | 72 | 22 |
| Unloading | Dry | 3 | P1 | 83.2 | 79.2 | 47.83 | 7.88 | 76 | 28 |
| Unloading | Dry | 4 | P1 | 83.5 | 79.5 | 48.03 | 7.78 | 66 | 26 |
| Unloading | Dry | 5 | P1 | 83.6 | 79.6 | 48.23 | 7.68 | 79 | 25 |
| Unloading | Dry | 6 | P1 | 84.6 | 80.6 | 48.43 | 7.58 | 79 | 28 |
| Unloading | Dry | 7 | P1 | 85 | 81 | 48.63 | 7.48 | 79 | 28 |
| Unloading | Dry | 8 | P1 | 85 | 81 | 48.83 | 7.38 | 70 | 30 |
| Unloading | Dry | 9 | P1 | 85 | 81 | 49.03 | 7.28 | 72 | 22 |
| Unloading | Dry | 10 | P1 | 85.3 | 81.3 | 49.23 | 7.18 | 76 | 28 |
| Unloading | Dry | 11 | P1 | 85.3 | 81.3 | 49.43 | 7.08 | 66 | 26 |
| Unloading | Dry | 12 | P1 | 85.3 | 81.3 | 49.63 | 6.98 | 79 | 25 |
| Unloading | Dry | 13 | P1 | 85.6 | 81.6 | 49.83 | 6.88 | 79 | 28 |
| Unloading | Dry | 14 | P1 | 85.7 | 81.7 | 50.03 | 6.78 | 79 | 28 |
| Unloading | Dry | 15 | P1 | 85.7 | 81.7 | 50.23 | 6.68 | 70 | 30 |
| Unloading | Dry | 1 | P2 | 85.9 | 81.9 | 76.02 | 16.14 | 72 | 22 |
| Unloading | Dry | 2 | P2 | 86 | 82 | 76.32 | 16.24 | 76 | 28 |
| Unloading | Dry | 3 | P2 | 86 | 82 | 76.62 | 16.34 | 66 | 26 |
| Unloading | Dry | 4 | P2 | 86 | 82 | 76.92 | 16.44 | 79 | 25 |
| Unloading | Dry | 5 | P2 | 86.2 | 82.2 | 77.22 | 16.54 | 79 | 28 |
| Unloading | Dry | 6 | P2 | 86.5 | 82.5 | 77.52 | 16.64 | 79 | 28 |
| Unloading | Dry | 7 | P2 | 86.5 | 82.5 | 77.82 | 16.74 | 70 | 30 |
| Unloading | Dry | 8 | P2 | 86.8 | 82.8 | 78.12 | 16.84 | 72 | 22 |
| Unloading | Dry | 9 | P2 | 87.1 | 83.1 | 78.42 | 16.94 | 76 | 28 |
| Unloading | Dry | 10 | P2 | 87.3 | 83.3 | 78.72 | 17.04 | 66 | 26 |
| Unloading | Dry | 11 | P2 | 87.3 | 83.3 | 79.02 | 17.14 | 79 | 25 |
| Unloading | Dry | 12 | P2 | 87.9 | 83.9 | 79.32 | 17.24 | 79 | 28 |
| Unloading | Dry | 13 | P2 | 87.9 | 83.9 | 79.62 | 17.34 | 79 | 28 |
| Unloading | Dry | 14 | P2 | 87.9 | 83.9 | 79.92 | 17.44 | 72 | 22 |
| Unloading | Dry | 15 | P2 | 88.1 | 84.1 | 80.22 | 17.54 | 76 | 28 |
| Unloading | Moisture | 1 | P3 | 82.4 | 78.4 | 3.58 | 3.6 | 66 | 26 |
| Unloading | Moisture | 2 | P3 | 82.4 | 78.4 | 3.57 | 3.59 | 79 | 25 |
| Unloading | Moisture | 3 | P3 | 82.5 | 78.5 | 3.56 | 3.58 | 79 | 28 |
| Unloading | Moisture | 4 | P3 | 82.5 | 78.5 | 3.55 | 3.57 | 79 | 28 |
| Unloading | Moisture | 5 | P3 | 82.9 | 78.9 | 3.54 | 3.56 | 72 | 22 |
| Unloading | Moisture | 6 | P3 | 83 | 79 | 3.53 | 3.55 | 76 | 28 |
| Unloading | Moisture | 7 | P3 | 83.1 | 79.1 | 3.52 | 3.54 | 66 | 26 |
| Unloading | Moisture | 8 | P3 | 83.1 | 79.1 | 3.51 | 3.53 | 79 | 25 |
| Unloading | Moisture | 9 | P3 | 83.2 | 79.2 | 3.5 | 3.52 | 79 | 28 |
| Unloading | Moisture | 10 | P3 | 83.4 | 79.4 | 3.49 | 3.51 | 79 | 28 |
| Unloading | Moisture | 11 | P3 | 83.5 | 79.5 | 3.48 | 3.5 | 72 | 22 |
| Unloading | Moisture | 12 | P3 | 83.6 | 79.6 | 3.47 | 3.49 | 76 | 28 |
| Unloading | Moisture | 13 | P3 | 84 | 80 | 3.46 | 3.48 | 66 | 26 |
| Unloading | Moisture | 14 | P3 | 84.1 | 80.1 | 3.45 | 3.47 | 79 | 25 |
| Unloading | Moisture | 15 | P3 | 84.6 | 80.6 | 3.44 | 3.46 | 79 | 28 |
| Unloading | Moisture | 1 | P4 | 84.8 | 80.8 | 4.45 | 5.4 | 79 | 28 |
| Unloading | Moisture | 2 | P4 | 85 | 81 | 4.46 | 5.41 | 72 | 22 |
| Unloading | Moisture | 3 | P4 | 85.2 | 81.2 | 4.47 | 5.42 | 76 | 28 |
| Unloading | Moisture | 4 | P4 | 85.2 | 81.2 | 4.48 | 5.43 | 66 | 26 |
| Unloading | Moisture | 5 | P4 | 86.1 | 82.1 | 4.49 | 5.44 | 79 | 25 |
| Unloading | Moisture | 6 | P4 | 86.3 | 82.3 | 4.5 | 5.45 | 79 | 28 |
| Unloading | Moisture | 7 | P4 | 86.4 | 82.4 | 4.51 | 5.46 | 79 | 28 |
| Unloading | Moisture | 8 | P4 | 86.5 | 82.5 | 4.52 | 5.47 | 72 | 22 |
| Unloading | Moisture | 9 | P4 | 86.6 | 82.6 | 4.53 | 5.48 | 76 | 28 |
| Unloading | Moisture | 10 | P4 | 86.6 | 82.6 | 4.54 | 5.49 | 66 | 26 |
| Unloading | Moisture | 11 | P4 | 86.9 | 82.9 | 4.55 | 5.5 | 79 | 25 |
| Unloading | Moisture | 12 | P4 | 87.3 | 83.3 | 4.56 | 5.51 | 79 | 28 |
| Unloading | Moisture | 13 | P4 | 87.3 | 83.3 | 4.57 | 5.52 | 79 | 28 |
| Unloading | Moisture | 14 | P4 | 87.3 | 83.3 | 4.58 | 5.53 | 76 | 28 |
| Unloading | Moisture | 15 | P4 | 87.3 | 83.3 | 4.59 | 5.54 | 66 | 26 |
| Cleaning | Dry | 1 | P5 | 88.8 | 84.8 | 13.28 | 6.58 | 72 | 22 |
| Cleaning | Dry | 2 | P5 | 89 | 85 | 13.08 | 6.48 | 83 | 30 |
| Cleaning | Dry | 3 | P5 | 89.2 | 85.2 | 12.88 | 6.38 | 83 | 30 |
| Cleaning | Dry | 4 | P5 | 89.2 | 87.7 | 12.68 | 6.28 | 76 | 28 |
| Cleaning | Dry | 5 | P5 | 89.2 | 87.7 | 12.48 | 6.18 | 76 | 28 |
| Cleaning | Dry | 6 | P5 | 89.7 | 87.8 | 12.28 | 6.08 | 71 | 28 |
| Cleaning | Dry | 7 | P5 | 89.7 | 87.9 | 12.08 | 5.98 | 59 | 24 |
| Cleaning | Dry | 8 | P5 | 89.7 | 87.9 | 11.88 | 5.88 | 72 | 22 |
| Cleaning | Dry | 9 | P5 | 89.7 | 88 | 11.68 | 5.78 | 83 | 30 |
| Cleaning | Dry | 10 | P5 | 89.7 | 88 | 11.48 | 5.68 | 83 | 30 |
| Cleaning | Dry | 11 | P5 | 89.9 | 88 | 11.28 | 5.58 | 76 | 28 |
| Cleaning | Dry | 12 | P5 | 90 | 88.1 | 11.08 | 5.48 | 76 | 28 |
| Cleaning | Dry | 13 | P5 | 90 | 88.4 | 10.88 | 5.38 | 71 | 28 |
| Cleaning | Dry | 14 | P5 | 90 | 88.5 | 10.68 | 5.28 | 59 | 24 |
| Cleaning | Dry | 15 | P5 | 90.1 | 88.5 | 10.48 | 5.18 | 72 | 22 |
| Cleaning | Dry | 1 | P6 | 91.7 | 88.7 | 13.48 | 14.64 | 83 | 30 |
| Cleaning | Dry | 2 | P6 | 91.7 | 88.8 | 13.68 | 14.74 | 83 | 30 |
| Cleaning | Dry | 3 | P6 | 91.7 | 88.8 | 13.88 | 14.84 | 76 | 28 |
| Cleaning | Dry | 4 | P6 | 91.8 | 90.9 | 14.08 | 14.94 | 76 | 28 |
| Cleaning | Dry | 5 | P6 | 91.9 | 91 | 14.28 | 15.04 | 71 | 28 |
| Cleaning | Dry | 6 | P6 | 91.9 | 91 | 14.48 | 15.14 | 59 | 24 |
| Cleaning | Dry | 7 | P6 | 91.9 | 91.1 | 14.68 | 15.24 | 72 | 22 |
| Cleaning | Dry | 8 | P6 | 92 | 91.3 | 14.88 | 15.34 | 83 | 30 |
| Cleaning | Dry | 9 | P6 | 92 | 91.4 | 15.08 | 15.44 | 83 | 30 |
| Cleaning | Dry | 10 | P6 | 92 | 91.4 | 15.28 | 15.54 | 76 | 28 |
| Cleaning | Dry | 11 | P6 | 92 | 91.4 | 15.48 | 15.64 | 76 | 28 |
| Cleaning | Dry | 12 | P6 | 92.1 | 91.5 | 15.68 | 15.74 | 71 | 28 |
| Cleaning | Dry | 13 | P6 | 92.4 | 91.6 | 15.88 | 15.84 | 59 | 24 |
| Cleaning | Dry | 14 | P6 | 92.5 | 91.6 | 16.08 | 15.94 | 72 | 22 |
| Cleaning | Dry | 15 | P6 | 92.5 | 91.6 | 16.28 | 15.84 | 83 | 30 |
| Cleaning | Moisture | 1 | P7 | 89.1 | 85.1 | 9.37 | 2.6 | 83 | 30 |
| Cleaning | Moisture | 2 | P7 | 89.6 | 85.2 | 9.17 | 2.59 | 76 | 28 |
| Cleaning | Moisture | 3 | P7 | 89.6 | 85.2 | 8.97 | 2.58 | 76 | 28 |
| Cleaning | Moisture | 4 | P7 | 89.6 | 85.6 | 8.77 | 2.57 | 71 | 28 |
| Cleaning | Moisture | 5 | P7 | 89.6 | 85.6 | 8.57 | 2.56 | 59 | 24 |
| Cleaning | Moisture | 6 | P7 | 89.8 | 85.6 | 8.37 | 2.55 | 72 | 22 |
| Cleaning | Moisture | 7 | P7 | 90.2 | 85.6 | 8.17 | 2.54 | 83 | 30 |
| Cleaning | Moisture | 8 | P7 | 91.1 | 85.7 | 7.97 | 2.53 | 83 | 30 |
| Cleaning | Moisture | 9 | P7 | 91.7 | 85.7 | 7.77 | 2.52 | 76 | 28 |
| Cleaning | Moisture | 10 | P7 | 91.8 | 85.7 | 7.57 | 2.51 | 76 | 28 |
| Cleaning | Moisture | 11 | P7 | 91.9 | 85.7 | 7.37 | 2.5 | 71 | 28 |
| Cleaning | Moisture | 12 | P7 | 91.9 | 85.7 | 7.17 | 2.49 | 59 | 24 |
| Cleaning | Moisture | 13 | P7 | 92.1 | 85.8 | 6.97 | 2.48 | 72 | 22 |
| Cleaning | Moisture | 14 | P7 | 92.2 | 85.9 | 6.77 | 2.47 | 83 | 30 |
| Cleaning | Moisture | 15 | P7 | 92.5 | 86 | 6.57 | 2.46 | 83 | 30 |
| Cleaning | Moisture | 1 | P8 | 92.7 | 86 | 9.37 | 4.4 | 76 | 28 |
| Cleaning | Moisture | 2 | P8 | 92.8 | 86 | 9.57 | 4.41 | 76 | 28 |
| Cleaning | Moisture | 3 | P8 | 92.8 | 86.1 | 9.77 | 4.42 | 71 | 28 |
| Cleaning | Moisture | 4 | P8 | 94.9 | 86.2 | 9.97 | 4.43 | 59 | 24 |
| Cleaning | Moisture | 5 | P8 | 95 | 87.1 | 10.17 | 4.44 | 72 | 22 |
| Cleaning | Moisture | 6 | P8 | 95 | 87.7 | 10.37 | 4.45 | 83 | 30 |
| Cleaning | Moisture | 7 | P8 | 95.1 | 87.7 | 10.57 | 4.46 | 83 | 30 |
| Cleaning | Moisture | 8 | P8 | 95.3 | 87.8 | 10.77 | 4.47 | 76 | 28 |
| Cleaning | Moisture | 9 | P8 | 95.4 | 87.9 | 10.97 | 4.48 | 76 | 28 |
| Cleaning | Moisture | 10 | P8 | 95.4 | 87.9 | 11.17 | 4.49 | 71 | 28 |
| Cleaning | Moisture | 11 | P8 | 95.4 | 87.9 | 11.37 | 4.5 | 59 | 24 |
| Cleaning | Moisture | 12 | P8 | 95.5 | 88 | 11.57 | 4.51 | 76 | 28 |
| Cleaning | Moisture | 13 | P8 | 95.6 | 88.1 | 11.77 | 4.52 | 76 | 28 |
| Cleaning | Moisture | 14 | P8 | 95.6 | 88.2 | 11.97 | 4.53 | 76 | 28 |
| Cleaning | Moisture | 15 | P8 | 95.6 | 88.5 | 12.17 | 4.54 | 71 | 28 |
| Expedition | Dry | 1 | P9 | 81.9 | 76.9 | 72.44 | 8.08 | 69 | 29 |
| Expedition | Dry | 2 | P9 | 82.1 | 77.1 | 72.14 | 7.98 | 79 | 28 |
| Expedition | Dry | 3 | P9 | 82.5 | 77.5 | 71.84 | 7.88 | 68 | 28 |
| Expedition | Dry | 4 | P9 | 82.8 | 78.5 | 71.54 | 7.78 | 55 | 24 |
| Expedition | Dry | 5 | P9 | 82.8 | 79.2 | 71.24 | 7.68 | 50 | 28 |
| Expedition | Dry | 6 | P9 | 83.2 | 79.5 | 70.94 | 7.58 | 69 | 29 |
| Expedition | Dry | 7 | P9 | 84.2 | 79.8 | 70.64 | 7.48 | 55 | 24 |
| Expedition | Dry | 8 | P9 | 84.2 | 80.2 | 70.34 | 7.38 | 69 | 29 |
| Expedition | Dry | 9 | P9 | 84.2 | 80.2 | 70.04 | 7.28 | 79 | 28 |
| Expedition | Dry | 10 | P9 | 84.7 | 80.4 | 69.74 | 7.18 | 68 | 28 |
| Expedition | Dry | 11 | P9 | 84.8 | 80.5 | 69.44 | 7.08 | 55 | 24 |
| Expedition | Dry | 12 | P9 | 85.2 | 81.2 | 69.14 | 6.98 | 50 | 28 |
| Expedition | Dry | 13 | P9 | 85.8 | 81.2 | 68.84 | 6.88 | 69 | 29 |
| Expedition | Dry | 14 | P9 | 85.9 | 81.3 | 68.54 | 6.78 | 55 | 24 |
| Expedition | Dry | 15 | P9 | 86.1 | 82.4 | 68.24 | 6.68 | 69 | 29 |
| Expedition | Dry | 1 | P10 | 86.1 | 82.5 | 81.04 | 16.14 | 79 | 28 |
| Expedition | Dry | 2 | P10 | 87.1 | 82.6 | 81.34 | 16.24 | 68 | 28 |
| Expedition | Dry | 3 | P10 | 87.1 | 82.6 | 81.64 | 16.34 | 55 | 24 |
| Expedition | Dry | 4 | P10 | 87.2 | 82.7 | 81.94 | 16.44 | 50 | 28 |
| Expedition | Dry | 5 | P10 | 87.2 | 83.2 | 82.24 | 16.54 | 69 | 29 |
| Expedition | Dry | 6 | P10 | 87.6 | 83.3 | 82.54 | 16.64 | 55 | 24 |
| Expedition | Dry | 7 | P10 | 87.6 | 84 | 82.84 | 16.74 | 69 | 29 |
| Expedition | Dry | 8 | P10 | 87.6 | 85 | 83.14 | 16.84 | 79 | 28 |
| Expedition | Dry | 9 | P10 | 87.6 | 85.1 | 83.44 | 16.94 | 68 | 28 |
| Expedition | Dry | 10 | P10 | 89.8 | 85.1 | 83.74 | 17.04 | 55 | 24 |
| Expedition | Dry | 11 | P10 | 89.8 | 85.1 | 84.04 | 17.14 | 50 | 28 |
| Expedition | Dry | 12 | P10 | 89.9 | 85.1 | 84.34 | 17.24 | 69 | 29 |
| Expedition | Dry | 13 | P10 | 89.9 | 87.1 | 84.64 | 17.34 | 55 | 24 |
| Expedition | Dry | 14 | P10 | 90.1 | 88.6 | 84.94 | 17.44 | 69 | 29 |
| Expedition | Dry | 15 | P10 | 90.1 | 89.4 | 85.24 | 17.54 | 79 | 28 |
| Expedition | Moisture | 1 | P11 | 78.7 | 74 | 67.15 | 3.6 | 68 | 28 |
| Expedition | Moisture | 2 | P11 | 79.3 | 74.5 | 66.85 | 3.59 | 55 | 24 |
| Expedition | Moisture | 3 | P11 | 79.3 | 75.2 | 66.55 | 3.58 | 50 | 28 |
| Expedition | Moisture | 4 | P11 | 79.6 | 75.5 | 66.25 | 3.57 | 69 | 29 |
| Expedition | Moisture | 5 | P11 | 79.6 | 75.9 | 65.95 | 3.56 | 55 | 24 |
| Expedition | Moisture | 6 | P11 | 81.3 | 75.9 | 65.65 | 3.55 | 69 | 29 |
| Expedition | Moisture | 7 | P11 | 81.3 | 76 | 65.35 | 3.54 | 79 | 28 |
| Expedition | Moisture | 8 | P11 | 81.7 | 76.2 | 65.05 | 3.53 | 68 | 28 |
| Expedition | Moisture | 9 | P11 | 81.8 | 76.3 | 64.75 | 3.52 | 55 | 24 |
| Expedition | Moisture | 10 | P11 | 81.8 | 76.4 | 64.45 | 3.51 | 50 | 28 |
| Expedition | Moisture | 11 | P11 | 82 | 76.9 | 64.15 | 3.5 | 69 | 29 |
| Expedition | Moisture | 12 | P11 | 82.1 | 77 | 63.85 | 3.49 | 55 | 24 |
| Expedition | Moisture | 13 | P11 | 82.1 | 77.9 | 63.55 | 3.48 | 69 | 29 |
| Expedition | Moisture | 14 | P11 | 82.2 | 78.6 | 63.25 | 3.47 | 79 | 28 |
| Expedition | Moisture | 15 | P11 | 82.4 | 78.9 | 62.95 | 3.46 | 68 | 28 |
| Expedition | Moisture | 1 | P12 | 82.5 | 79 | 80.1 | 5.4 | 55 | 24 |
| Expedition | Moisture | 2 | P12 | 82.6 | 79 | 79.8 | 5.41 | 50 | 28 |
| Expedition | Moisture | 3 | P12 | 82.9 | 79.2 | 79.5 | 5.42 | 69 | 29 |
| Expedition | Moisture | 4 | P12 | 83.8 | 79.3 | 79.2 | 5.43 | 55 | 24 |
| Expedition | Moisture | 5 | P12 | 84 | 80 | 78.9 | 5.44 | 69 | 29 |
| Expedition | Moisture | 6 | P12 | 84.2 | 80.6 | 78.6 | 5.45 | 79 | 28 |
| Expedition | Moisture | 7 | P12 | 84.7 | 82.5 | 78.3 | 5.46 | 68 | 28 |
| Expedition | Moisture | 8 | P12 | 84.8 | 82.7 | 78 | 5.47 | 55 | 24 |
| Expedition | Moisture | 9 | P12 | 84.8 | 83 | 77.7 | 5.48 | 50 | 28 |
| Expedition | Moisture | 10 | P12 | 84.8 | 83.1 | 77.4 | 5.49 | 69 | 29 |
| Expedition | Moisture | 11 | P12 | 85.5 | 84.2 | 77.1 | 5.5 | 55 | 24 |
| Expedition | Moisture | 12 | P12 | 85.9 | 84.5 | 76.8 | 5.51 | 69 | 29 |
| Expedition | Moisture | 13 | P12 | 85.9 | 84.9 | 76.5 | 5.52 | 79 | 28 |
| Expedition | Moisture | 14 | P12 | 86.1 | 85.5 | 76.2 | 5.53 | 68 | 28 |
| Expedition | Moisture | 15 | P12 | 86.1 | 87.6 | 75.9 | 5.54 | 55 | 24 |
